# Neurotransmitter-Triggered Transfer of Exosomes Mediates Oligodendrocyte–Neuron Communication

**DOI:** 10.1371/journal.pbio.1001604

**Published:** 2013-07-09

**Authors:** Carsten Frühbeis, Dominik Fröhlich, Wen Ping Kuo, Jesa Amphornrat, Sebastian Thilemann, Aiman S. Saab, Frank Kirchhoff, Wiebke Möbius, Sandra Goebbels, Klaus-Armin Nave, Anja Schneider, Mikael Simons, Matthias Klugmann, Jacqueline Trotter, Eva-Maria Krämer-Albers

**Affiliations:** 1 Department of Molecular Cell Biology, University of Mainz, Mainz, Germany; 2 Focus Program Translational Neuroscience, University of Mainz, Mainz, Germany; 3 Max Planck Institute of Experimental Medicine, Göttingen, Germany; 4 Department of Molecular Physiology, University of Saarland, Homburg, Germany; 5 Department of Psychiatry and Psychotherapy, University of Göttingen, German Center for Neurodegenerative Diseases, DZNE Goettingen, Cluster of Excellence Nanoscale Microscopy and Molecular Physiology of the Brain (EXC171) and DFG Research Center of Molecular Physiology of the Brain, Göttingen, Germany; 6 Department of Neurology, University of Göttingen, Göttingen, Germany; 7 Translational Neuroscience Facility, Department of Physiology, School of Medical Sciences, University of New South Wales, Sydney, Australia; Stanford University School of Medicine, United States of America

## Abstract

Reciprocal interactions between neurons and oligodendrocytes are not only crucial for myelination, but also for long-term survival of axons. Degeneration of axons occurs in several human myelin diseases, however the molecular mechanisms of axon-glia communication maintaining axon integrity are poorly understood. Here, we describe the signal-mediated transfer of exosomes from oligodendrocytes to neurons. These endosome-derived vesicles are secreted by oligodendrocytes and carry specific protein and RNA cargo. We show that activity-dependent release of the neurotransmitter glutamate triggers oligodendroglial exosome secretion mediated by Ca^2+^ entry through oligodendroglial NMDA and AMPA receptors. In turn, neurons internalize the released exosomes by endocytosis. Injection of oligodendroglia-derived exosomes into the mouse brain results in functional retrieval of exosome cargo in neurons. Supply of cultured neurons with oligodendroglial exosomes improves neuronal viability under conditions of cell stress. These findings indicate that oligodendroglial exosomes participate in a novel mode of bidirectional neuron-glia communication contributing to neuronal integrity.

## Abstract 2

Neuronal activity provokes myelinating oligodendrocytes to release exosomes by stimulation of ionotropic glutamate receptors, and that once released, these vesicles are internalized by neurons conveying neuroprotection.

## Author Summary

Brain function largely depends on the communication between electrically excitable neurons and surrounding glial cells. Myelinating oligodendrocytes are a type of brain cell that insulate major neuronal processes (axons) and help to sustainably maintain axonal health, which is poorly understood in molecular terms. Several cell types release microvesicles termed exosomes that include genetic information (primarily RNA) and can act as vehicles transferring specific cargo to target cells. Here, we demonstrate that exosomes secreted by oligodendrocytes in response to neuronal signals enter neurons to make their cargo functionally available to the neuronal metabolism. We revealed in cultured cells that exosome release from oligodendrocytes is triggered by the neurotransmitter glutamate through activation of ionotropic glutamate receptors. We also show that glial exosomes are internalized by neurons via an endocytic pathway. By modifying oligodendroglial exosomes with a reporter enzyme, we could demonstrate that the exosome cargo is recovered by target neurons in culture as well as *in vivo* after injection of exosomes into the mouse brain. Neurons challenged with stressful growth conditions were protected when treated with oligodendroglial exosomes. The study introduces a new concept of reciprocal cell communication in the nervous system and identifies the signal-mediated transfer of exosomes from oligodendrocytes to neurons contributing to the preservation of axonal health.

## Introduction

In the CNS, oligodendrocytes insulate axons with a multilayered myelin sheath enabling rapid impulse conduction. Formation of functional axon-myelin units depends on bidirectional axon-glia interaction [1],[2]. During nervous system development neuronal signals including activity-dependent neurotransmitter release control the differentiation of oligodendrocytes and myelination [3]–[5]. Axon-glia communication remains important throughout life. In addition to axon ensheathment, oligodendrocytes provide trophic support to neurons critical for long-term axonal integrity [6]. Glial support has been suggested to represent an ancestral function independent of myelination [7]. The mechanisms of neuron-glia communication essential to sustainably maintain and protect the highly specialized axon-glial entity over a lifetime are not well understood. Recent studies indicate that glycolytic oligodendrocytes provide axons with external energy substrates such as lactate [8],[9]. These studies reveal new insights into axonal energy supply, although it remains still open how other resources (such as enzymes of a certain half-life) reach distal sites of axons.

Oligodendrocytes release membrane vesicles with the characteristics of exosomes, which include specific myelin proteins such as proteolipid protein (PLP) [10],[11]. Since exosomes have the capacity to affect neighboring cells, they have been generally implicated in intercellular communication [12],[13] Exosomes of 50–100 nm in size are generated within the endosomal system and secreted upon fusion of multivesicular bodies (MVBs) with the plasma membrane. The exosomal membrane exhibits the topology of the plasma membrane and encloses cytoplasmic cargo. Most if not all cell types secrete exosomes and other microvesicles, budding from the plasma membrane. Consequently, body fluids such as serum, urine, and CSF contain significant amounts of mixed microvesicles, including exosomes [14]. Exosomes carry cell-type-specific components as well as common cargo, including proteins involved in MVB biogenesis, heat shock proteins, and integral membrane proteins such as integrins and tetraspanins. Furthermore, exosomes contain mRNA and miRNA, which upon horizontal transfer can alter protein expression, thus modulating the properties of recipient cells [15]–[17]. They have been described to contribute to immune responses, to spread pathogens such as viruses and prions, to modulate the tumor cell micro-environment, and furthermore to educate the phenotype of bone marrow cells [18]–[20]. Although cells exhibit a basal level of release, secretion of exosomes is a regulated process. Increase in cytoplasmic Ca^2+^ triggers exosome release from several cell types, including neurons and oligodendrocytes [10],[21],[22].

In this study, we analyze the role of exosomes in axon-glia communication. We demonstrate that neuronal activity-mediated release of the neurotransmitter glutamate regulates oligodendroglial exosome secretion by activation of glial ionotropic glutamate receptors. In turn, neurons internalize exosomes released from oligodendrocytes and retrieve their cargo. Furthermore, our results indicate that oligodendrocyte-derived exosomes mediate neuroprotective functions. These findings reveal a novel mode of cell communication among cells of the CNS that may be employed by oligodendrocytes to support axons.

## Results

### Oligodendroglial Cre Driver Mice Exhibit Reporter Gene Recombination in Neurons

**Figure 1:**
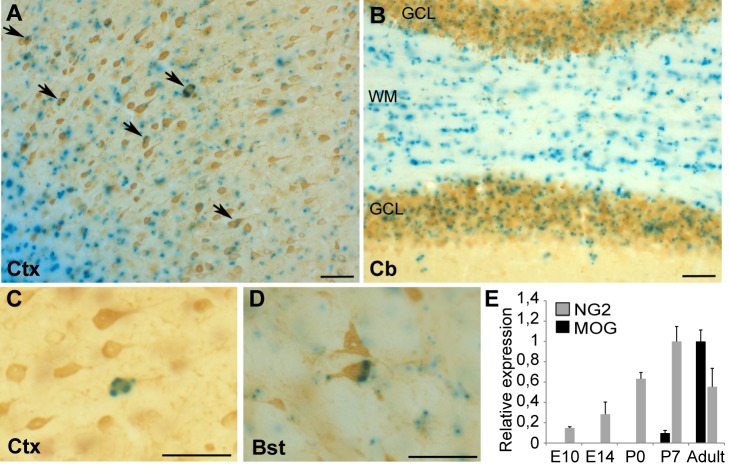
Neuronal recombination in MOGi-Cre/lacZ reporter mice. (A–D) Immunohistochemical analysis of brain sections derived from MOGi-Cre/Rosa26-LacZ reporter mice. β-galactosidase positive cells were stained using X-gal as substrate and neurons were labeled with antibodies against NeuN. Recombined cells were detected in the cortex (Ctx, A, C), cerebellum (Cb, B), and brainstem (Bst, D). Scale bar, 50 µm. (E) MOG and NG2 expression analysis by q-PCR of E10, E14, P0, P7, and adult total brains (*n* = 3). The maximal expression was set to 1. NG2 is shown as an example of a gene expressed in progenitor cells.

Expression of Cre recombinase under control of a cell-type-specific promoter is utilized to drive the recombination of floxed target genes in a defined subset of cells within a tissue. MOGi-Cre mice carry Cre as a knock-in allele under control of the endogenous MOG promoter, which is described to be specifically active in the late stage of oligodendrocyte maturation [23] driving Cre expression in oligodendrocytes exclusively [24],[25]. However, analysis of double transgenic MOGi-Cre/Rosa26-lacZ mice revealed reporter gene expression not only in oligodendrocytes but also in a subset of neurons in several brain regions (Figure 1). In the cerebellar granule cell layer, 17% of NeuN-labeled cells were positive for LacZ, while a lower number of recombined cells carrying neuronal markers were present in the cortex (3.8%), hippocampus (1.2%), and brainstem (2.9%). This finding may be explained by (1) activity of the MOG promoter in individual neurons or their precursors or (2) the horizontal transfer of Cre recombinase from oligodendrocytes to neurons. By q-PCR, MOG transcripts were either undetectable or at the detection limit in the embryonic brain and turned up during the first postnatal week coinciding with the appearance of mature oligodendrocytes (Figure 1E). Therefore, it is unlikely that MOG-promoter activity in early embryonic progenitor cells is responsible for recombination persisting through neuronal differentiation into adulthood. The present study explores the possibility of horizontal transfer of molecules from oligodendrocytes to neurons by vesicles secreted from MVBs via the exosome pathway.

### Oligodendrocyte MVBs Are Present at Periaxonal Sites

**Figure 2:**
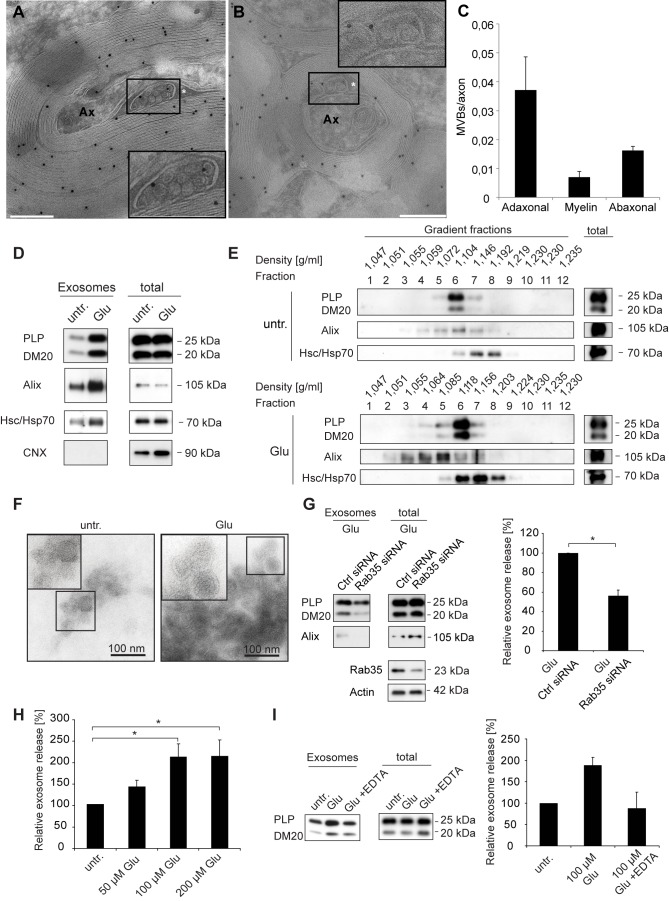
Adaxonal localization of MVBs and glutamate-dependent release of exosomes. (A–C) Immuno-electron microscopy analysis of cross-sections of myelinated axons in the optic nerve or spinal cord of adult mice labeled with antibodies against PLP (scale bar, 250 nm, asterisk marks adaxonal loop). Insets depict enlarged views of a MVB in the oligodendrocyte adaxonal cytoplasmic loop (A) and a fusion profile indicating the release of vesicles with the size corresponding to exosomes (B). (C) Quantification of MVBs located adaxonal, abaxonal, and within compact myelin in optic nerves. MVB number was normalized per axon. (D) Western blot analysis of PLP, Alix, Hsc/Hsp70, and calnexin (CNX) in cell lysates and exosomes pelleted by differential centrifugation from culture supernatants of primary oligodendrocytes (pOL) treated with glutamate (Glu, 100 µM) for 5 h or untreated (untr.). (E) Density gradient analysis of exosomes purified from supernatants of pOL treated with 100 µM glutamate versus untreated control cells. (F) Electron microscopic analysis of 100,000× g exosome pellets derived from supernatants collected over a period of 5 h from untreated (left image) or glutamate-treated cells (100 µM). Scale bar, 100 nm. (G) Transfection of pOL with Rab35- or control-siRNA (Ctrl) and quantification of glutamate-dependent exosome release. Western blot signals of exosomal PLP were normalized to total cellular PLP and defined as relative exosome release (*n* = 5). (H) Administration of 50 (*n* = 5), 100 (*n* = 8), 200 µM (*n* = 10) glutamate to pOL, and quantitative analysis of exosome release. (I) Incubation of pOL with the Ca^2+^-chelator EDTA and quantification of glutamate-dependent exosome release (*n* = 3). Error bars, SEM (* *p*<0.05; Wilcoxon test).

Exosomes are generated by inward budding of the endosomal limiting membrane and stored in MVBs before release. Ultrastructural examination of optic nerve or spinal cord myelinated fibers by electron microscopy revealed that MVBs are present in the cytoplasm of oligodendrocytes, including the innermost uncompacted wrapping of the myelin membrane (adaxonal loop) in close proximity to the axon (Figure 2A). In rare cases, we detected fusion profiles of MVBs indicating the release of intraluminal vesicles (exosomes) into the periaxonal space (Figure 2B). The MVBs occasionally carried immunolabeling of LAMP1 (not shown) or PLP in the MVB limiting membrane and the intraluminal vesicles. The quantification revealed that MVBs are most prominent in the adaxonal loop at periaxonal sites, compared to their localization in the outer abaxonal loop or in channels between myelin lamellae (Figure 2C). Among adaxonal MVBs, 29±8.8% carried immunolabeling for PLP.

Taken together, these *in vivo* observations support the concept of exosomes playing a role in oligodendrocyte-neuron communication.

### Glutamate Stimulates Oligodendroglial Exosome Release

We first asked whether oligodendroglial exosome secretion is regulated by neuronal signals. Our previous work revealed that elevation of cytosolic Ca^2+^ levels stimulates exosome release [10]. Since neurotransmitters such as glutamate mediate Ca^2+^ signaling in oligodendrocytes [26], we investigated whether glutamate triggers oligodendroglial exosome release. Primary cultured oligodendrocytes were treated with glutamate for 5 h and exosomes were isolated from the supernatant by differential centrifugation. Oligodendroglial exosomes are known to include the myelin tetraspan protein PLP and its splice variant DM20 [10], which was utilized to identify oligodendrocyte-derived exosomes by Western blotting. The amount of PLP/DM20 detected in 100,000× g pellets obtained from supernatants of glutamate-treated cells was significantly increased compared to untreated cells, while total PLP expression in the cells was unaffected. Consistently, we observed higher levels of the exosomal marker proteins Alix and Hsc/Hsp70 in the 100,000× g pellet obtained from glutamate stimulated cells, indicating that more exosomes were secreted (Figure 2D). Previous studies showed that oligodendrocyte viability is affected by glutamate-mediated toxicity depending on intensity and duration of glutamate exposure as well as other side effects such as oxidative stress [27],[28]. We observed no apparent damage of cultured oligodendrocytes 5 h after glutamate administration (Figure S1A). To finally rule out that membrane fragments released from dying cells contaminated the exosome preparation, we investigated the integrity of the plasma membrane by evaluating lactate dehydrogenase (LDH) release and propidium iodide exclusion and found no detrimental influence of glutamate within the exosome collection period (Figure S1B and S1C). Moreover, we utilized the ER-localized protein calnexin to determine contamination with nonexosomal membranes, which were virtually absent in most preparations (Figure 2D).

We further purified exosomes by sucrose density gradient centrifugation and detected PLP, Alix, and Hsc/Hsp70 in fractions of the typical density range reported for exosomes. Compared to untreated cells, the amounts of PLP, Alix, and Hsc/Hsp70 were increased, indicating that more exosomes had been isolated from glutamate-treated cultures (Figure 2E). The slightly different position of the exosomal marker proteins in the gradient may reflect different exosome subpopulations, consistent with the current view of exosome heterogeneity [12]. Nanoparticle tracking analysis revealed that glutamate-stimulated cells release significantly more particles with a mean size of 95 nm, reflecting the expected size of exosomes (Figure S1D). Notably, the size distribution of the released particles was not influenced by glutamate treatment. Examination of 100,000× g pellets derived from supernatants of glutamate-stimulated cells by electron microscopy identified larger aggregates of vesicles with the characteristic size and structure of exosomes as compared to pellets obtained from control cells, which released only few particles within the 5 h collection period (Figure 2F).

To obtain further proof that glutamate acts on exosome secretion, we performed siRNA silencing of the small GTPase Rab35. Previous work has demonstrated that Rab35 regulates exosome secretion from oligodendrocytes [29]. Silencing of Rab35 in primary oligodendrocytes (by 60±6.8% on the protein level) interfered with the glutamate-dependent release of PLP and Alix demonstrating a specific effect of glutamate on exosome secretion (Figures 2G and S1E). Thus, the particles released from oligodendrocytes in response to glutamate are released in a Rab35-dependent manner, and match the marker profile, size, and the density of exosomes.

Next, we determined the dose dependence of glutamate-mediated exosome release. A concentration of 50 µM was sufficient to stimulate exosome release and saturation was reached at the dose of 100 µM (Figure 2H). Moreover, we asked if extracellular Ca^2+^ is mobilized to stimulate glutamate-dependent exosome release and pre-incubated primary oligodendrocytes with EDTA, a nonmembrane permeable chelator of divalent cations, before exposure to glutamate. Depletion of divalent ions from the medium completely prevented stimulation of exosome release by glutamate, indicating that entry of extracellular Ca^2+^ through ionotropic glutamate channels is essential to trigger exosome release (Figure 2I). Analysis of intracellular Ca^2+^ levels using a fluorescent calcium indicator showed that the cells responded to glutamate with a rise in intracellular calcium (Figure S1F). Taken together, these results demonstrate that the neurotransmitter glutamate triggers Ca^2+^-dependent exosome release from oligodendrocytes.

### Glial NMDA and AMPA Receptors Mediate Exosome Release

**Figure 3:**
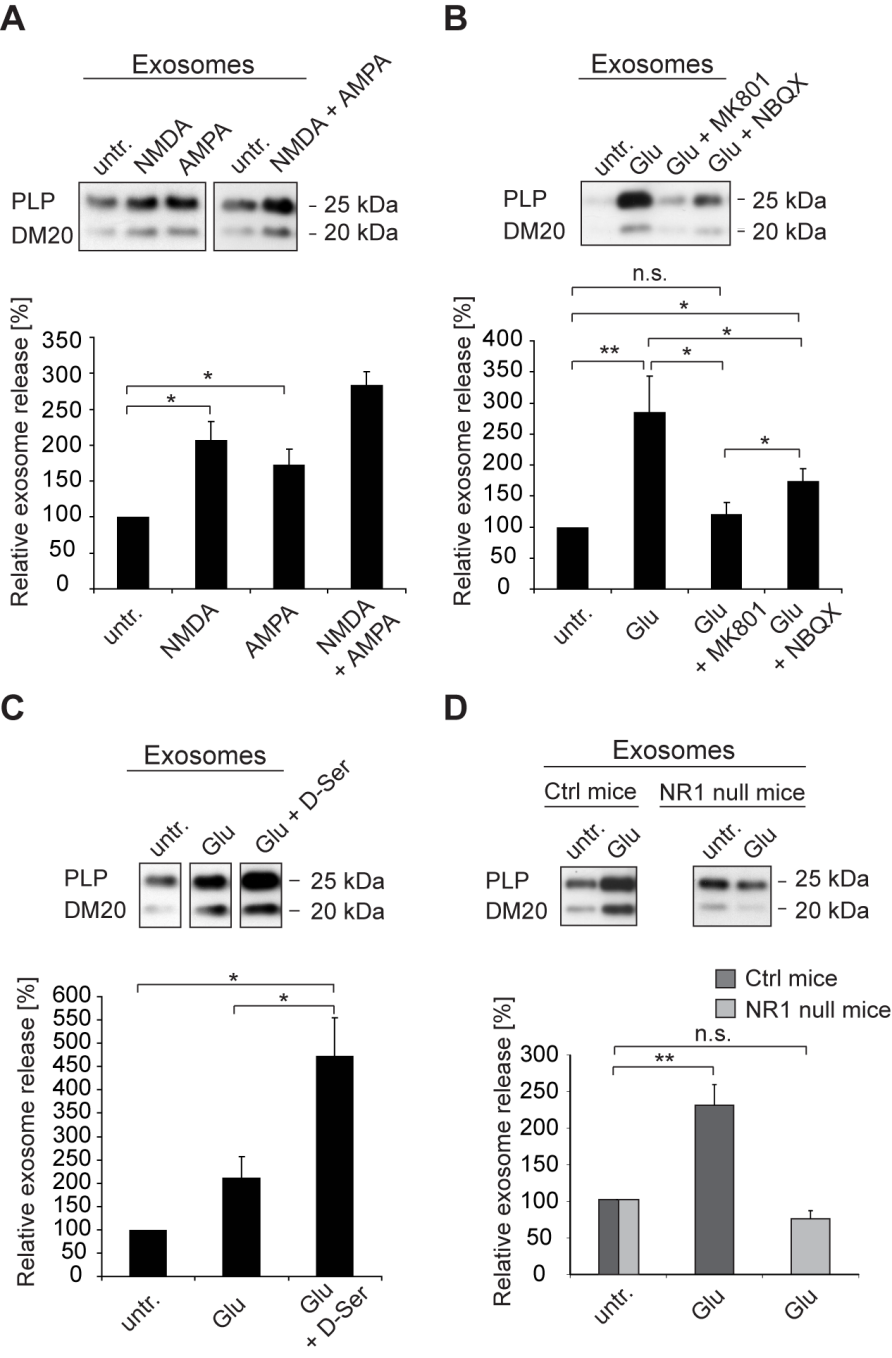
NMDA and AMPA receptors mediate exosome release. Quantification of exosome release upon (A) stimulation with glutamate receptor agonists NMDA (100 µM, *n* = 7), AMPA (100 µM, *n* = 5), or NMDA and AMPA (*n* = 4), (B) application of NMDA and AMPA receptor antagonists MK801 (5 µM, *n* = 9) or NBQX (25 µM, *n* = 9), or (C) the NMDA receptor co-agonist D-Serine (100 µM, *n* = 5). (D) Glutamate-stimulated exosome release from NMDA receptor-deficient oligodendrocytes obtained from CNP^Cre/+^*NR1^flox/flox^ mice (*n* = 6) compared to CNP^Cre/+^ control mice (*n* = 9). Representative Western blots of PLP in exosome pellets are depicted. Error bars, SEM (n.s., not significant ; * *p*<0.05; ** *p*<0.01; Wilcoxon test).

Glutamate-mediated Ca^2+^ influx across the oligodendroglial plasma membrane occurs through ligand-operated Ca^2+^ channels, such as NMDA and AMPA receptors [26]. Expression of functional receptors and their subunits by mature oligodendrocytes and their precursors has been described previously [30]–[33]. We confirmed their presence in differentiated oligodendrocytes *in vitro* by immunocytochemistry, with the expected localization of NMDA receptors in the membrane sheets, while AMPA receptors appear preferentially on cell bodies and primary processes (Figure S2A, B) [34]. To investigate if these receptors regulate oligodendroglial exosome release, we stimulated cells with receptor-specific agonists to provoke Ca^2+^ entry. NMDA as well as AMPA application induced a significant increase in exosome secretion. Moreover, administration of both agonists had a synergistic effect (Figure 3A). Next, we co-applied glutamate with selective NMDA and AMPA receptor antagonists. MK801 and NBQX block oligodendroglial NMDA and AMPA receptors, respectively [32]. Both antagonists impaired glutamate-dependent exosome release (Figure 3B), though NMDA receptor inhibition affected exosome release more potently. When we applied the known NMDA receptor co-agonist D-serine [35] together with glutamate, D-serine potentiated glutamate-dependent exosome release over glutamate alone (Figure 3C).

To obtain further evidence for the role of NMDA receptors in oligodendroglial exosome secretion, we used conditional knock-out mice lacking the essential NMDA receptor subunit NR1 selectively in oligodendrocytes (CNP^+/Cre^*NR1^flox/flox^) [36],[37]. The floxed NR1-locus is recombined by Cre, which is expressed under control of the CNP promoter. Conditional NR1-null mice were heterozygous for ROSA26-flox-stop-EYFP, which we used as reporter for Cre expressing cells. As expected, primary cultured oligodendrocytes derived from these mice expressed EYFP confirming efficient recombination in oligodendrocytes (unpublished data). Furthermore, NR1 was absent from mature oligodendrocytes expressing PLP (Figure S2A). We compared the relative exosome release in response to glutamate in cells lacking NR1 (CNP^+/Cre^*NR1^flox/flox^) and in control cells (CNP^+/Cre^*NR1^+/+^) and found that exosome release was not stimulated by glutamate in the absence of NR1 (Figure 3D). These results suggest that NMDA receptors are essential for the glutamate-dependent secretion of exosomes from oligodendrocytes.

### Neuronal Activity Stimulates Glial Exosome Release

**Figure 4:**
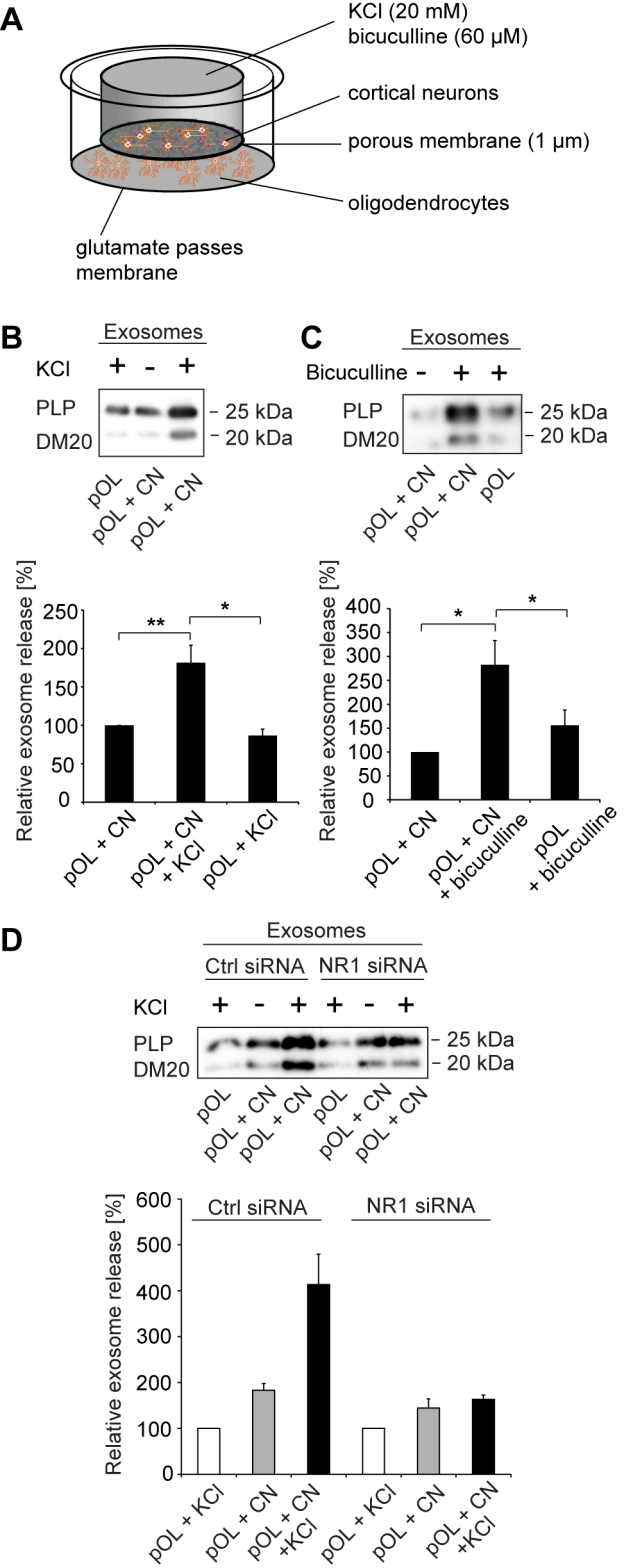
Neuronal activity stimulates exosome release from oligodendrocytes. (A) Cartoon illustrating the Boyden chamber co-culture of cortical neurons (CN) and pOL separated by a membrane permitting exchange of metabolites and small particles (<1 µm). (B) Application of 20 mM KCl (*n* = 9) or (C) 60 µM bicuculline (*n* = 6) to CN in the top well and analysis of relative exosome release from pOL. KCl or bicuculline applied to pOL is depicted as control (*n* = 5). (D) Boyden chamber co-culture of CN and pOL transfected with siRNA against NR1 or control siRNA. Depolarization of CN with 20 mM KCl and quantification of oligodendroglial exosome release (*n* = 3). Error bars, SEM (* *p*<0.05; ** *p*<0.01; Wilcoxon test).

Electrically active axons releasing glutamate evoke oligodendroglial Ca^2+^ signals [38]–[40]. To test if oligodendroglial exosome secretion is linked to neuronal electrical activity, we made use of a transwell device (Boyden chamber), allowing contact-free co-culture of cells and exchange of metabolites by diffusion through pores (Figure 4A). Cortical neurons grown for 7 d *in vitro* and placed on top of oligodendrocytes were depolarized with 20 mM potassium to trigger neurotransmitter release. Depolarization induced a significant increase in exosome secretion from oligodendrocytes (Figure 4B). Exposure of oligodendrocytes to potassium in the absence of neurons was ineffective. In addition, we treated cortical neurons grown for 14 d to allow synapse formation with the GABA_A_-receptor antagonist bicuculline to block inhibitory activity and enhance spontaneous glutamatergic activity [22]. Again, oligodendroglial exosome release was strongly increased in response to enhanced neuronal electrical activity (Figure 4C). Although bicuculline provoked a response of isolated oligodendrocytes, the stimulation of exosome release was 3 times more potent in the presence of neurons. To investigate if glutamate released by neurons acts on oligodendroglial NMDA receptors, we interfered with NR1 expression using RNAi. Silencing of NR1 in oligodendrocytes reduced the amount of secreted exosomes upon neuronal depolarization (Figures 4D and S2C). We obtained similar results using oligodendrocytes derived from conditional NR1 null mice (Figure S2D). These findings suggest that glutamate released by neurons in response to depolarization activates oligodendroglial glutamate receptors (mainly of the NMDA receptor subtype) stimulating exosome secretion. Thus, oligodendroglial exosome release is linked to neuronal electrical activity.

### Neurons Internalize Glial Exosomes

**Figure 5:**
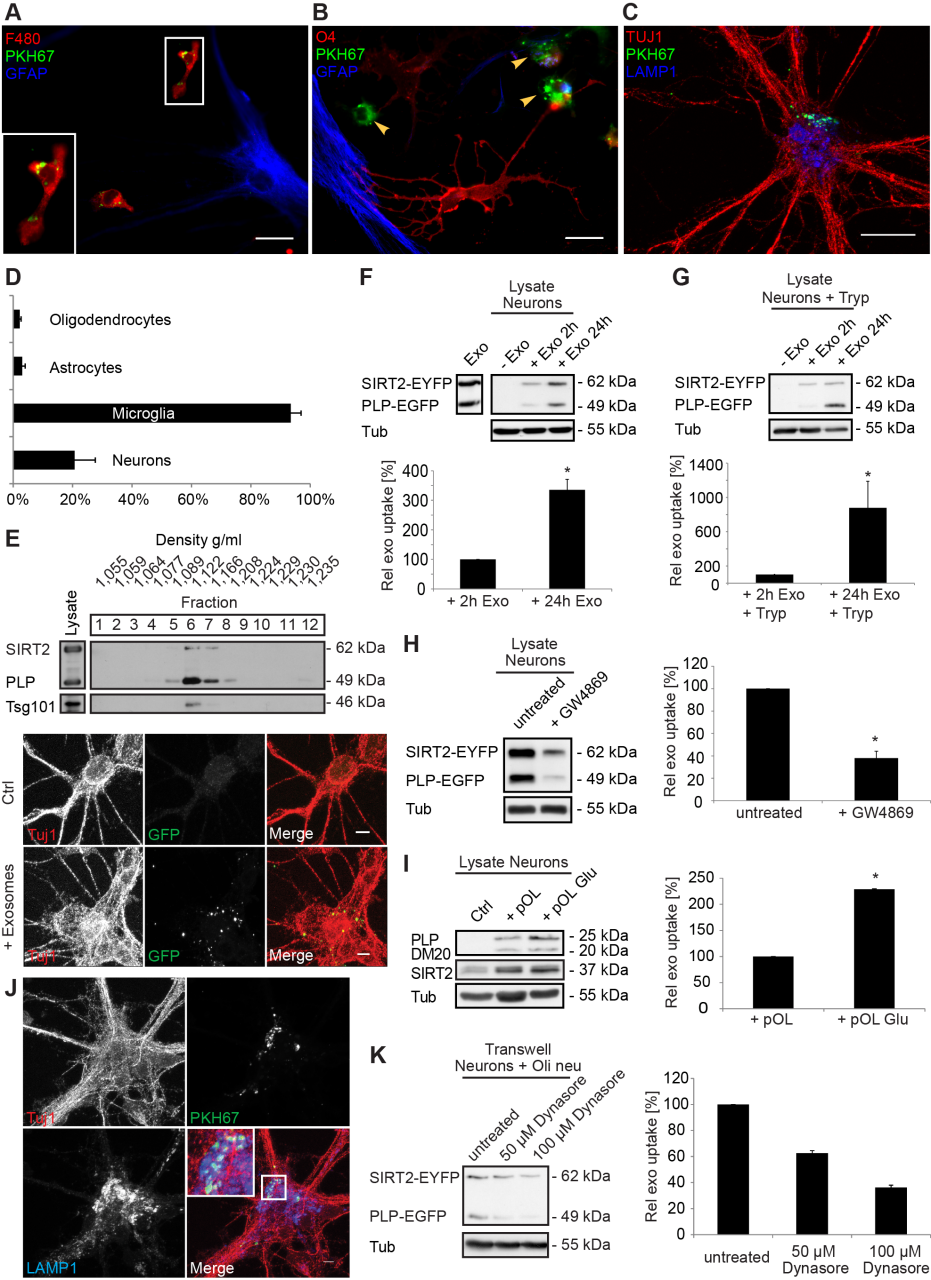
Primary cortical neurons internalize oligodendroglial exosomes. (A–D) pOL were stained with the lipophilic dye PKH67 (green), washed, and subsequently co-cultured in Boyden chambers for 2 d with mixed neural cultures containing astrocytes (A, B, blue marker GFAP), oligodendrocytes (B, red marker O4), and microglia (A, red marker F4/80, B, arrowheads) or with CN (C, red marker Tuj1). Scale bar, 20 µm. (D) Quantification of exosome uptake by the different types of target cells. Error bars, SEM, (*n* = 3). (E) Fluorescent exosomes containing SIRT2-EYFP and PLP-EGFP were purified by sucrose density gradient centrifugation from Oli-neu cells and co-incubated with CN for 24 h. The Western blot depicts EGFP and the exosomal marker Tsg101 in gradient fractions. Images show maximum projections of confocal Z-stacks of Tuj1-stained neurons after incubation with exosomes (scale bar, 5 µm). (F) Western blots of purified Oli-neu exosomes (input, left lane) and neuronal lysates after treatment with exosomes (Exo). EGFP/EYFP depicts exosome markers, Tubulin (Tub) is used as normalization standard. Relative exosome uptake reflects normalized signals of SIRT2-EYFP and PLP-EGFP associated with neuronal lysates (*n* = 8). (G) To remove surface-bound exosomes, neurons were treated with trypsin (Tryp) before lysis (*n* = 5). (H–K) Boyden chamber co-culture of oligodendroglial cells and CN for 2–3 d and analysis of exosomal PLP and SIRT2 in neurons by Western blot (H, I, and K) or immunostaining (J). (H) PLP-EGFP and SIRT2-EYFP expressing Oli-neu cells were treated or not with 5 µM GW4869 inhibiting exosome release (*n* = 6). (I) pOL were treated with 100 µM glutamate stimulating exosome release (*n* = 5). (J) Co-culture of CN with PKH67-labelled (green) pOL and immunostaining of CN with Tuj1 (red) and the late endosomal/lysosomal marker LAMP1 (blue). Maximum projection of a confocal Z-stack. Scale bar, 5 µm. (K) Neurons were pre-treated with Dynasore and co-cultured for 1 d with Oli-neu cells releasing SIRT2-EYFP and PLP-EGFP labeled exosomes (*n* = 4). Error bars, SEM (* *p*<0.05; Wilcoxon-test).

We further studied the fate of the released exosomes and asked whether exosomes can be internalized by other neural cells. We utilized the transwell co-culture system and cultured oligodendrocytes labeled with the fluorescent dye PKH67 on porous filters, allowing the passage of exosomes. PKH67 is a lipophilic dye that is released in association with exosomes [41]. Cortical neurons or glial cultures were placed in the bottom chamber and imaged to visualize the uptake of fluorescent membrane particles in different neural cell types. We observed internalization of oligodendroglia-derived particles by 20.7±7% of the cortical neurons and by 93.4±3.5% of the microglia, while uptake by astrocytes and oligodendrocytes was detected only in 3±1% and 2.2±0.4% of the cells, respectively (Figure 5A–D, see Figure S3 for overview). These data suggest that extracellular vesicles including exosomes released by oligodendrocytes are internalized preferentially by microglia and neurons.

To confirm exosome uptake by neurons, we exposed primary cortical neurons to density gradient purified exosomes derived from the oligodendroglial cell line Oli-neu ectopically expressing SIRT2-EYFP and PLP-EGFP. Both proteins are sorted to oligodendroglial exosomes (Figure 5E) [10]. PLP-EGFP is located in the exosomal membrane, while SIRT2-EYFP is intraluminal. Incubation of neurons with these exosomes led to an uptake of fluorescent particles (Figure 5E). Furthermore, neurons incubated with purified exosomes accumulated glial-specific, exosome-associated PLP and SIRT2 with time (Figure 5F). Exosomes were indeed internalized, since removal of surface-bound exosomes by protease treatment before the lysis did not prevent the detection of PLP-EGFP/SIRT2-EYFP in neurons (Figure 5G). Transfer of exosomes can also be visualized after co-culture of primary cortical neurons with Oli-neu cells in Boyden chambers. We utilized the neutral sphingomyelinase inhibitor GW4869, which inhibits the release of exosomes [42]. Application of GW4869 to Oli-neu cells reduced the amount of secreted and thus internalized exosomes significantly, verifying that exosomes were responsible for the horizontal transfer of proteins and not other cell-derived particles (Figure 5H). Moreover, the transfer appears to be selective, since untagged EGFP overexpressed in Oli-neu cells is not delivered to neurons (Figure S4).

We further employed primary oligodendrocytes to examine exosome transfer to neurons. Exosome-associated PLP/DM20 as well as SIRT2 were detectable in neurons co-cultured with oligodendrocytes in Boyden chambers (Figure 5I). Stimulation of oligodendroglial exosome release with glutamate significantly increased the level of PLP/DM20 and SIRT2 associated with neurons, demonstrating that neuronal uptake correlates with the amount of exosomes. To identify the subcellular destination of internalized exosomes, we performed double-labeling experiments of PKH67-stained glial exosomes together with endocytic markers. PKH67-stained exosomes accumulated within the neurons in endosome-like structures partly overlapping with LAMP1-positive late endosomes/lysosomes (Figure 5J). A 3D reconstruction of confocal images confirmed that exosomes were located inside the neurons (Figure S5A). To exclude that excess dye instead of stained exosomes was taken up by neurons, we pelleted PKH67-labelled exosomes and incubated neurons with exosomes and with exosome-depleted supernatant, respectively. PKH67-positive particles only associated with neurons when the exosome pellet was used, ruling out a transfer of excess dye (unpublished data).

To determine whether neurons internalize oligodendroglial exosomes by endocytosis, we pretreated neurons with Dynasore and Pitstop-2 inhibiting dynamin and clathrin-dependent endocytosis, respectively [43],[44]. Dynasore and Pitstop2 application, as well as inhibition of actin polymerization by Cytochalasin D treatment, reduced the neuronal uptake of oligodendroglial exosomes (Figures 5K and S5B,C). Impeding cholesterol-dependent (clathrin-independent) endocytosis by Methyl-β-Cyclodextrin application, which sequesters cholesterol from the plasma membrane, did not affect the uptake (Figure S5C). Importantly, oligodendroglial cells secreted normal levels of exosomes in the presence of the inhibitors (Figure S5D). Similar results were obtained utilizing the neuronal cell line HT22 (Figure S5E–G). Expression of dominant negative dynamin K44A in HT22 cells inhibited endocytosis of Transferrin-Alexa568 and uptake of exosomes (Figure S5H–J). These results indicate that neurons internalize oligodendroglial exosomes via an endocytic pathway that requires dynamin, clathrin, and actin polymerization.

### Retrieval of Exosome Cargo by Neurons

**Figure 6:**
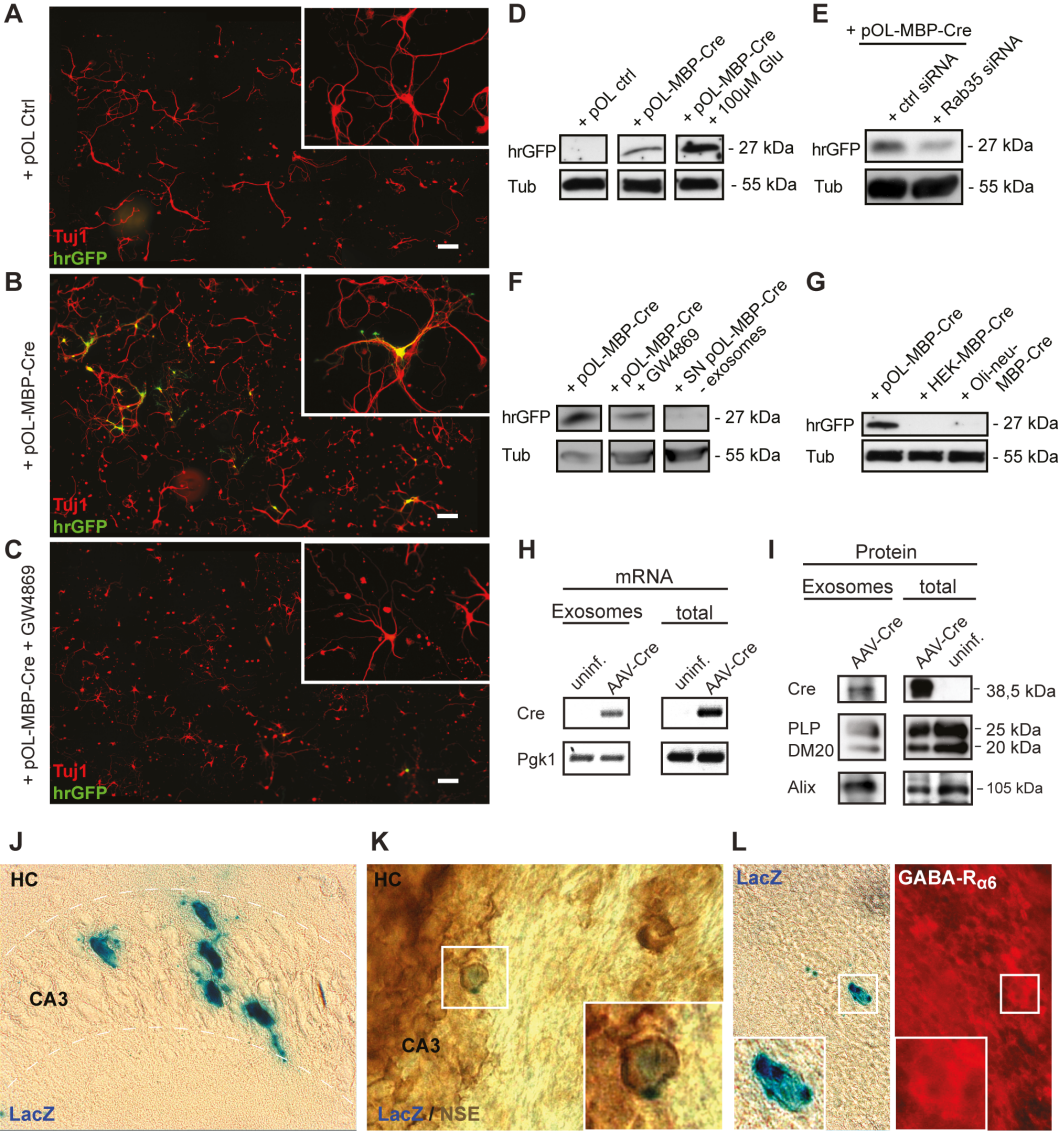
Functional retrieval of exosomal cargo by neurons. (A–G) Boyden chamber co-culture of pOL transduced by recombinant AAV/MBP-Cre (pOL-MBP-Cre) and CN transduced by AAV/CBA-floxstop-hrGFP reporter vector. hrGFP expression indicates Cre-mediated recombination. Tuj1 staining (red) of CN exposed to (A) nontransduced pOL, (B) pOL-MBP-Cre, or (C) pOL-MBP-Cre treated with 5 µM GW4869. Scale bar, 100 µm. (D–G) Reporter gene expression (hrGFP) in target neurons detected by Western blotting of neuronal lysates. CN were exposed to (D) pOL-MBP-Cre treated or not with 100 µM glutamate, (E) transfected with Rab35 or control siRNA, or (F) treated or not with 5 µM GW4869. (F, right lane) Exposure of CN to exosome-depleted culture supernatant from pOL-MBP-Cre does not lead to reporter gene expression in neurons. (G) pOL, Oli-neu cells, or HEK cells transduced with AAV/MBP-Cre co-cultured with CN. (H) Cre RT-PCR and (I) Western blot of exosomes derived from pOL-MBP-Cre or control cells. Pgk1 (mRNA), PLP/DM20, and Alix (protein) are shown as standards. (J–L) Stereotactic injection of exosomes derived from pOL-MBP-Cre into the (J, K) hippocampus (HC) and (L) cerebellum of ROSA26-lacZ reporter mice. β-galactosidase positive cells were stained using X-gal and neurons were stained for (K) neuron-specific enolase (NSE) or for (L) GABA-R_α6_. Mice (*n* = 5) were analyzed 14 d after injection.

Targeting of oligodendroglial exosomes to endosomes and late endosomes in neurons may result in lysosomal degradation or the recovery of exosomal components. In addition to protein cargo, oligodendroglial exosomes include distinct RNA species (Figure S6). To analyze, if the exosomal cargo is functionally retrieved by neurons, we employed Cre-mediated recombination as a reporter. Primary oligodendrocytes were infected with a replication-deficient recombinant Adeno-associated virus (AAV) vector encoding Cre-recombinase under control of the oligodendrocyte-specific MBP promoter (AAV/MBP-Cre) prior to co-culture with neurons in transwells [45]. These neurons had been transduced with reporter virus delivering the ubiquitous chicken β-actin promoter, followed by a floxed transcriptional termination element and the GFP coding region (AAV/CBA-floxstop-GFP). Reporter protein expression in neurons is specifically induced upon Cre-mediated excision of the floxed sequence [46]. Indeed, neurons acquired GFP reporter expression upon co-culture with AAV/MBP-Cre–infected oligodendrocytes (Figure 6A,B). Inhibition of glial exosome secretion by the sphingomyelinase inhibitor GW4869 (Figure 6C,F) or siRNA-mediated knockdown of Rab35 (Figure 6E) interfered with neuronal GFP expression, while stimulation of oligodendroglial exosome release with glutamate enhanced reporter expression (Figure 6D). Neurons incubated with oligodendroglial culture supernatant depleted from exosomes by 100,000× g centrifugation did not acquire GFP expression (Figure 6F). Furthermore, co-culture of reporter-virus–infected neurons and AAV/MBP-Cre–infected HEK cells or Oli-neu cells, which do not synthesize MBP promoter-driven Cre, did not result in GFP expression (Figure 6G), demonstrating that Cre expression in the donor cells is required and excluding a potential leakiness of the viral expression system. Consistent with these results, Cre protein, as well as Cre mRNA, were detectable in exosomes pelleted from supernatants of AAV/MBP-Cre–infected oligodendrocytes (Figure 6H,I). Taken together, these results demonstrate that the cargo of glial exosomes internalized by neurons is functionally retrieved within the target cells.

To provide the proof of principle that exosome transfer to neurons can occur *in vivo*, exosomes derived from AAV/MBP-Cre–infected oligodendrocytes were stereotactically injected into the cerebellum and hippocampus of adult Rosa26-lacZ reporter mice. Exosome internalization and cargo retrieval labels target cells as β-galactosidase-positive cells. In the injected hippocampus, individual neurons within the pyramidal cell layer of the CA3 region were positive for β-gal and neuron-specific enolase (eight cells per injection, *n* = 5, Figure 6J,K). In the cerebellum, we observed single recombined cells, which carried the GABA-R_α6_ subunit, a marker of cerebellar granule cells (five cells per injection, *n* = 5, Figure 6L). The number of detected recombined neurons may be limited by spatial restraints of the injected exosomes and by the fact that the retrieval of Cre from exosomes requires several steps before recombination can take place. Recombined cells were only present in animals injected with Cre-exosomes and never detected in brains of control mice injected with glial exosomes lacking Cre or at the contra-lateral side of injection. These experiments demonstrate that oligodendroglial exosomes can be internalized by neurons *in vivo* and validate the concept that exosome-mediated transfer of Cre from oligodendrocytes to neurons may underlie the neuronal reporter gene recombination observed in oligodendrocyte-specific Cre-driver mice (Figure 1).

### Exosome Uptake Occurs at Axonal and Somatodendritic Sites

**Figure 7:**
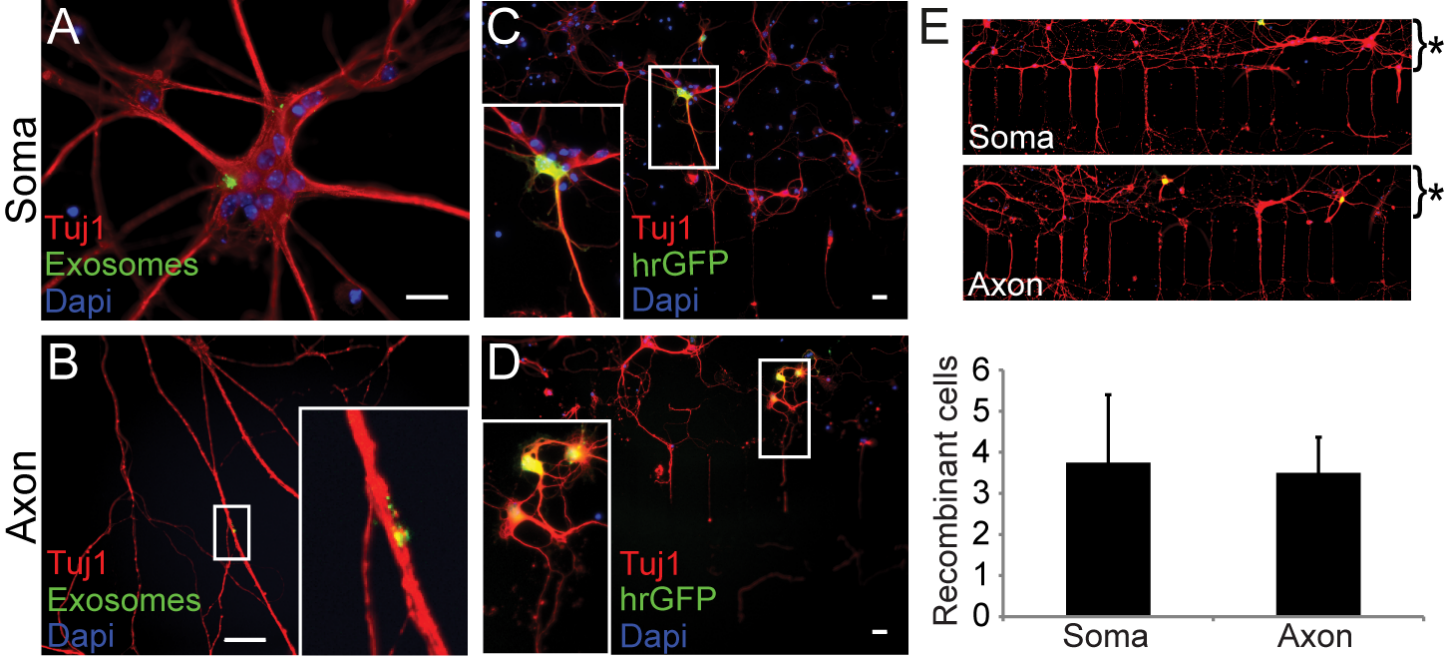
Somatodendritic and axonal uptake of exosomes. CN were cultured in microfluidic chambers and axons were allowed to grow through the microgrooves for 7 d. Exosomes isolated from PKH67 stained (A, B green) or AAV/MBP-Cre infected pOL (C, D) were applied to either the somatodendritic (A, C) or axonal (B, D) compartment of the device. Cre-containing exosomes were added to AAV/CBA-floxstop-hrGFP infected CN. hrGFP expression indicates Cre-mediated recombination (C, D green). CN were stained with Tuj1 (red) and nuclei with Dapi (blue). Scale bar, 20 µm. (E) Quantification of recombinant neurons located within 100 µm above the microgrooves. Exemplary pictures are depicted above the chart. Asterisk indicates the area of quantification. Error bars, SEM (*n* = 4).

We utilized microfluidic chambers to examine whether exosomes are internalized at the somatodendritic or the axonal domain. Cortical neurons cultured with their cell bodies in one compartment of the chamber grow axons through microgrooves into the other compartment of the chamber. Isolated exosomes labeled with PKH67 dye or containing Cre recombinase were added either to the somatodendritic or the axonal compartment and neuronal exosome uptake was monitored by imaging of exosomes or Cre reporter detection (Figure 7). Internalization of fluorescent exosomes was visible at the somatodendritic as well as at the axonal domain of the neurons. After application of Cre-bearing exosomes, we quantified the number of recombined neurons located within an area 100 µm from the microgrooves, thus focusing on neurons having the chance to project axons through the microgrooves into the axonal compartment (not all neuronal cell bodies in this area will successfully grow axons through the microgrooves). The number of recombined neurons did not differ significantly upon somatodendritic or axonal addition of exosomes. Thus, uptake of exosomes is possible at both sites.

### Oligodendroglial Exosomes Protect Neurons from Oxidative Stress and Starvation

**Figure 8:**
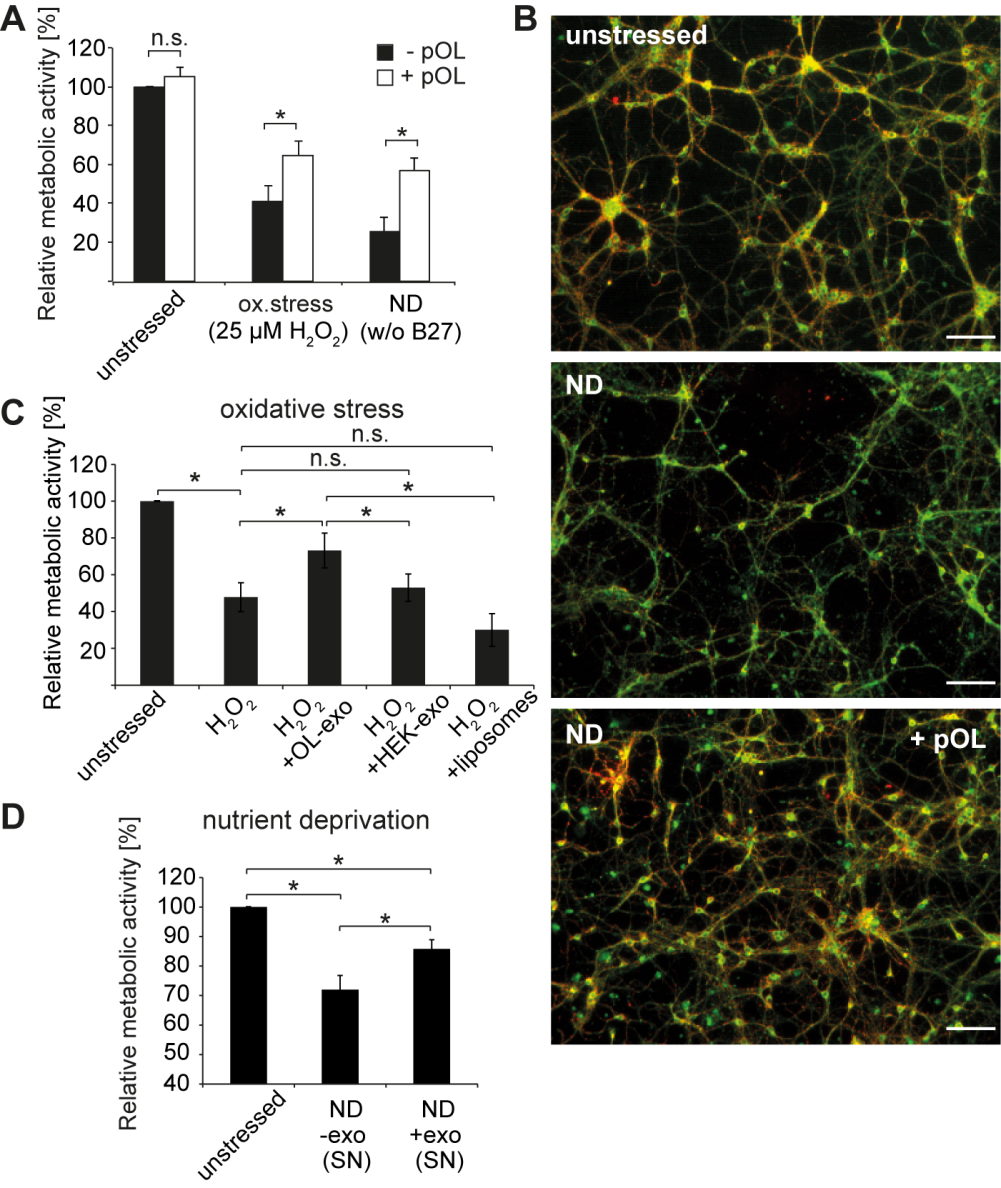
Neuroprotective role of oligodendroglial exosomes. (A) Boyden chamber co-culture of CN with or without pOL for 48 h under unstressed, oxidative stress, or nutrient deprivation (ND) conditions. Control CN cultured without pOL (black bars) were grown in oligodendrocyte-conditioned medium depleted from exosomes. Oxidative stress was induced after co-culture by challenging CN with 25 µM H_2_O_2_ for 1 h (*n* = 5). For nutrient deprivation, cells were cultured in medium lacking B27 supplement (*n* = 5). Neuronal viability was assayed by MTT assay. (B) Staining of the outer mitochondrial membrane with MitoCapture after nutrient deprivation and co-culture with and without pOL. Unstressed cells were grown in full medium without pOL. Scale bar, 100 µm. (C) Application of isolated exosomes and liposomes to neurons under conditions of cell stress. MTT assay of CN after single addition of isolated exosomes from pOL and HEK293T cells as well as liposomes 12–14 h prior to H_2_O_2_ stress (25 µM for 1 h) compared to unstressed or sham treated controls (*n* = 5). (D) MTT assay of CN subjected to nutrient deprivation and treated with exosome-containing (SN+exos) or exosome-deprived (SN w/o exos) oligodendrocyte culture supernatants for 12–14 h (*n* = 5). Error bars, SEM (n.s., not significant; * *p*<0.05; Wilcoxon-test).

To investigate if exosomes convey bioactivity to neurons, we performed viability assays on cultured neurons exposed to oligodendroglial exosomes. Oligodendrocytes and neurons were co-cultured in Boyden-chambers for 48 h, allowing a constant supply of the neurons with oligodendrocyte-derived factors including exosomes. Control cells were cultured without oligodendrocytes in oligodendrocyte pre-conditioned medium, deprived of exosomes but still containing secreted soluble factors. Subsequently, neurons were subjected to a MTT assay to measure their metabolic activity. Neurons grown under optimal conditions were not significantly affected by the presence of exosome-producing oligodendrocytes (Figure 8A, unstressed). Intriguingly, when neurons were subjected to stress conditions such as oxidative stress (25 µM H_2_O_2_ for 1 h) or nutrient deprivation (culture in absence of B27 supplement), their metabolic activity was significantly increased in the presence of exosome-secreting oligodendrocytes (Figure 8A). Neuronal metabolic activity was increased by 23.6±7.4% and 31.2±7% after oxidative stress exposure and nutrient deprivation, respectively. Oxidative stress was not alleviated when neurons were challenged by oxidative stress prior to co-culture (not shown), indicating that exosome supply is protective but is not sufficient to rescue damaged neurons. MitoCapture staining demonstrates that co-culture of nutrient-deprived neurons with exosome-producing oligodendrocytes prevents the breakdown of the mitochondrial membrane potential (Figure 8B).

In a second approach, we directly incubated neurons with isolated exosomes or exosome-containing supernatants and determined their metabolic activity compared to untreated neurons (Figure 8C,D). A single administration of isolated oligodendroglial exosomes 12–14 h prior to oxidative stress resulted in a significant increase in neuronal metabolic activity by 25.4±9.5%. In contrast, pretreatment of neurons with HEK293T-derived exosomes or artificial liposomes was ineffective, indicating a specific function of oligodendroglial exosomes. In the nutrient-deprivation paradigm, the metabolic activity of starving neurons increased by 13.8±3.1% upon incubation with oligodendrocyte-conditioned culture supernatant containing exosomes compared to supernatants deficient of exosomes (Figure 8D), while exosome-containing HEK cell supernatant was ineffective (not shown). A single application of isolated exosomes had only a minor supportive effect on the metabolic activity of starving neurons (unpublished data). However, the level of metabolic activity in starving neurons was clearly dependent on the presence of oligodendroglial exosomes. The results of these experiments demonstrate that oligodendrocyte-derived exosomes support the neuronal metabolism under conditions of cell stress, suggesting a role in neuroprotection.

## Discussion

**Figure 9:**
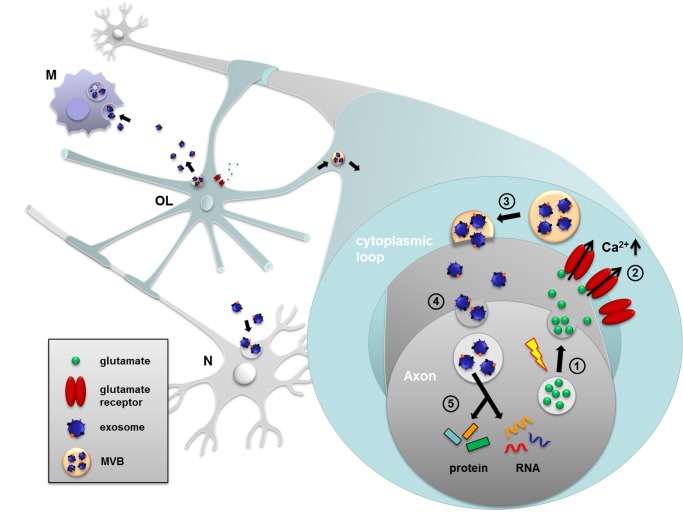
Oligodendroglial exosome secretion and its role in reciprocal neuron-glia communication. Electrically active axons release the neurotransmitter glutamate (1) inducing Ca^2+^-entry through oligodendroglial glutamate receptors (2). The rise in intracellular Ca^2+^ triggers exosome release from oligodendrocytes (OL) along internodes or at somas (3). In turn, exosomes are internalized by neurons (N) at axons or cell bodies (4) and their protein and RNA cargo is functionally retrieved (5). Exosome uptake by microglia (M) is also indicated.

The role of exosomes in cell communication and intercellular transfer of bioactive molecules has been recognized in tissues outside the nervous system [12],[13] and is best established among cells of the immune system, where exosomes have been demonstrated to modulate antigen presentation and the immune response [19]. Communication between neurons and myelinating oligodendrocytes is essential to form and maintain a fully functional axon-glial unit. Here, we demonstrate a novel mode of reciprocal neuron-glia communication involving neurotransmitter-regulated transfer of exosomes from oligodendrocytes to neurons. We present *in vit*ro and *in vivo* evidence for the uptake of glia-derived exosomes by neurons and, importantly, the functional recovery of their cargo. Furthermore, the supply of cultured neurons with oligodendroglial exosomes supports the neuronal metabolism and increases neuronal viability under conditions of cell stress. We propose a model where activity-triggered exosome release from myelinating oligodendrocytes occurs along internodes and paranodal regions into the periaxonal space, where they are internalized by axons (Figure 9). This concept is supported by the prevalence of MVBs in the inner mesaxon *in situ*, which appear to fuse with the glial plasma membrane and release exosomes into the periaxonal space. Nevertheless, release of oligodendroglial exosomes may also occur from cell bodies and their uptake also takes place at neuronal somas or dendrites. Thus, exosomal cargo can act at axonal or dendritic sites, which is likely to be associated with distinct functional outcomes.

### Glutamate-Dependent Stimulation of Exosome Release from Oligodendrocytes

Evidence exists for activity-dependent release of glutamate along axons of white matter tracts occuring by vesicular means [47],[48] and by reversal of Na^+^-dependent glutamate transporters [34],[49]. On the glial side, glutamate receptors of the NMDA subtype appear to be preferentially localized to the oligodendrocyte periphery including myelin and the periaxonal membrane [32],[33], where they are ideally positioned to sense axonal glutamate release. Our results demonstrate that glutamate triggers exosome release from differentiated oligodendrocytes by activating Ca^2+^ permeable glial NMDA and AMPA receptors. While the physiological role of glutamate signaling to oligodendrocytes is still under debate, overstimulation is known to damage oligodendrocytes in pathological conditions due to excessive Ca^2+^ signaling [34],[50],[51]. Thus, a concern associated with our findings was that glutamate exposure could induce cell death and lead to the unspecific release of membrane fragments. We had no indication of cell disintegration or cell death within the period of exosome collection. Moreover, particle release was also induced by glutamate receptor agonists, which alone are not sufficient to induce oligodendroglial cell death [52]. Several studies show that glutamate-mediated cell death depends on receptor-independent secondary parameters such as the action of glutamate transporters, complement exposure, or oxidative stress [27],[53],[54]. In addition, the particles released in response to glutamate meet all criteria of exosomes (homogeneous size below 100 nm, presence of ubiquitous and cell-type-dependent marker proteins, density) and particle release was dependent on extracellular Ca^2+^ and the GTPase Rab35, which have been shown to control oligodendroglial exosome secretion. Notably, exosomes released in constitutive and glutamate-stimulated fashion share similar biophysical characteristics, although it is possible that individual components differ.

Our results suggest that exosome release occurs in response to neuronal electrical activity. Neuronal impulses have been implicated in the control of oligodendrocyte differentiation and myelination [55]–[57]. Oligodendrocyte precursor cells as well as mature oligodendrocytes respond to the firing of neighboring neurons with Ca^2+^ signals, which is mediated by glutamatergic and purinergic signaling [4],[26],[40],[58]. It has been demonstrated that the release of glutamate from electrically active axons initiates translation of the major myelin protein MBP, thus promoting myelination [5]. Activity-stimulated exosome release may well accompany the myelination-promoting effects of neuronal impulses.

We observed a dominant role of the NMDA receptor subtype in the control of glutamate-dependent exosome release, while AMPA receptor activation also played a role. Oligodendrocytes lacking the obligatory NMDA receptor subunit NR1 could not be stimulated by glutamate to release exosomes. Glial NMDA receptors are characterized by a low degree of voltage-dependent Mg^2+^ block [31], which in neurons is released by AMPA receptor activation. It is possible that the role of AMPA-receptor signaling in exosome secretion is to reinforce Ca^2+^ influx through NMDA receptors by removal of Mg^2+^ ions from the channel pore. This could explain why AMPA receptor activation is sufficient to induce exosome release, while NMDA receptor activation appears necessary. Two recent studies have shown that the conditional deletion of oligodendroglial NR1 in mice does not affect oligodendrocyte differentiation and myelination [59],[60]. Assuming the transfer of exosomes to neurons and the contribution of their cargo to the neuronal metabolism, one might anticipate that NMDA receptor deficiency in oligodendrocytes results in axonal degeneration. However, since exosome secretion occurs in a constitutive mode, a basal level of release will be maintained in these mice. Thus, an influence on axonal integrity would probably only become apparent when these mice are exposed to conditions of stress.

### Internalization of Exosomes by Neurons

Neurons and microglia internalize oligodendroglial exosomes effectively, whereas uptake by oligodendrocytes and astrocytes occurs only sporadically. Microglia take up exosomes unspecifically by macropinocytosis [61], while internalization of oligodendroglial exosomes by neurons is mediated by selective clathrin- and dynamin-dependent endocytosis. As yet unknown receptors, mediating uptake of oligodendroglial exosomes, are likely to be present on neurons (or even on a subset of neurons). In contrast to neurons, which recover the exosomal cargo functionally, microglia appear to degrade the exosomes. It is unclear how the cargo is retrieved from the endosome to reach its site of action.

Injection of Cre-containing exosomes into the mouse brain provokes recombination in neurons, which involves functional Cre enzyme. This finding is consistent with the recombination of the reporter in neurons upon crossing of MOGi-Cre mice with Rosa26-lacZ mice, since Cre recombinase may be transferred via exosomes from neighboring oligodendrocytes. Only a minority of all neurons exhibits reporter activity in both cases, indicating that Cre packaging into exosomes or its retrieval in neurons is not very efficient. In the MOGi-Cre mice, however, we cannot fully exclude minimal MOG-promoter activity in early progenitors or mature neurons.

### Function of Glia to Neuron Exosome Transfer

Exosomes carry a multitude of molecules including proteins, lipids, and also RNA that potentially can affect target cells. In particular, the delivery of mRNAs and miRNAs by exosomes (and other types of extracellular vesicles) has been shown to modulate target cell functions [15]–[17],[41],[62],[63]. Our results suggest that oligodendroglial exosomes support the neuronal metabolism and exhibit neuroprotective functions. Neurons treated with oligodendroglial exosomes were less sensitive to oxidative stress or starvation. At present, we can only speculate about the nature of the neuroprotective activity. Since exosomes are complex molecular entities and carry multiple enzymes with functions in metabolism or relieve of oxidative stress ([10] and Table S1), it is possible that several components contribute to this function. One of these factors may be Hsc/Hsp70, which has a well-established role in neuroprotection and is known to be supplemented from adjacent glial cells [64]. Hsc/Hsp70 is sorted to oligodendroglial exosomes and also transferred to neurons. There is evidence in the literature that a transfer of molecules from glia to neurons takes place, but the mechanisms remain unknown. It has been shown that the squid giant axon receives Hsc/Hsp70 produced by periaxonal glial cells [65] and newly synthesized glial RNAs are delivered to the giant axon as a result of axonal depolarization [66]. Moreover, a classic study hypothesizes that voltage-dependent sodium channels are shuttled from Schwann cells to axons to replenish channels during their life cycle [67].

It is tempting to speculate that the exosome-dependent delivery of bioactive molecules contributes to the glial support of axons, which has been discovered in mice deficient in the myelin proteins PLP and 2′, 3′-cyclic nucleotide 3′-phosphodiesterase (CNP). These mice develop a secondary axonal degeneration despite the presence of morphologically normal myelin [2],[36],[68]. Two recent studies provide compelling evidence that after myelination, glycolytic oligodendrocytes support axons with energy metabolites such as lactate and furthermore, interference with oligodendroglial lactate export results in axonal degeneration [8],[9]. However, it remains less clear how lactate supply is conceptionally linked to the lack of PLP and CNP in the mouse models of glial support. Exosomes carry both PLP and CNP as well as a heterogeneous group of enzymes and their secretion occurs independent of myelination (as does glial support). It is possible that exosome transfer complements the energy supply of neurons by delivering metabolizing enzymes (see Table S1). The coupling of exosome transfer to axonal electrical activity would link this process to neuronal energy consumption. It will be interesting to analyze exosomes derived from PLP- and CNP-deficient oligodendrocytes with respect to their components, neuronal transfer capacity, and protective function. A vesicle-dependent pathway of axonal support may also exist in the PNS. It has been shown that myelinating Schwann cells supply injured and regenerating axons with ribosomal subunits in association with vesicles [69],[70].

### Neural Exosomes and Disease

Exosome-mediated intercellular communication may be a more widespread phenomenon in the CNS involved in diverse physiological functions and pathological conditions [71]–[73]. Neurons also have the capability to secrete exosomes in an activity-dependent fashion, which has been suggested to modulate synaptic plasticity [22]. Microglia release plasma-membrane-derived vesicles that modulate synaptic activity by increasing neurotransmission [74]. Under brain inflammatory conditions such as EAE or multiple sclerosis, the amount of these vesicles in the CSF is increased [75]. Exosomes may also have detrimental effects under disease conditions and it has been proposed that they propagate pathogenic proteins through the CNS [76]. Several pathogenic proteins involved in CNS diseases, such as prions [18], β-amyloid peptide [77], superoxide-dismutase [78], and α-synuclein [79] are released from cells in association with exosomes. Moreover, cellular cholesterol homeostasis appears to be regulated by exosome release [80]. Notably, there is accumulating evidence that exosomes can pass the blood brain barrier and thus are promising tools for diagnostic and therapeutic applications in the future [16],[81].

In conclusion, this study reveals a novel mode of bidirectional neuron-glia interaction that couples neuronal activity to the external supply of neurons with glia-derived bioactive molecules. We suggest a role of exosome transfer from glia to neurons in metabolic support of neurons and thus neuroprotection.

## Materials and Methods

### Ethics Statement

Experiments were in compliance with the animal policies of the University of Mainz, approved by the German Federal State of Rheinland Pfalz, in accordance with the European Community Council Directive of November 24, 1986 (86_609_EEC).

### Animals, Cell Culture, and AAV Infection

Mouse strains: (1) wild-type C57Bl/6N, (2) CNP^Cre/+^ [36], (3) oligodendroglial NR1 knockout mice CNP^+/Cre^*NR1^flox/flox^ [37]. Conditional NR1-null mice were also heterozygous for ROSA26-flox-stop-EYFP [82], which we used as reporter for Cre expressing cells. (4) MOG^+/Cre^*R26R^+/lacZ^ [24],[83].

Mice of either sex of the strain C57Bl/6N were used for preparation of primary oligodendrocyte (pOL) cultures. Primary oligodendrocytes, mixed glial cells, and the cell line Oli-neu were prepared and cultured in Sato 1% HS as described [84]. Purity of the cultures was assessed by regular quality controls (immunostaining) and varied between 80% and 95%. Contaminating cells were of astroglial (3%–15%), microglial (1%–5%), and neuronal (5%–10%) origin. Cortical neurons were prepared from E14 embryonic mice as described [85] and were essentially glia free with less than 1% of contaminating astrocytes. HEK293T cells were cultured in DMEM+10% FCS and 1 mM sodium pyruvate. HT22 cells were cultured in DMEM+10% FCS. rAAV plasmids contained the chicken β-actin promoter (CBA) followed by a transcriptional termination sequence and the cDNA encoding hrGFP [45],[46], or the 1.3 kb mouse MBP-promoter driving Cre-recombinase. The AAV cassettes containing the woodchuck hepatitis virus posttranscriptional regulatory element (WPRE) and the bovine growth hormone polyadenylation sequence (bGHpA) were flanked by AAV2 inverted terminal repeats. Mosaic AAV1/2 vectors were produced by transfection of HEK cells as described and purified by discontinuous iodixanol centrifugation [86]. Genomic titers were determined by PCR using primers against WPRE and vectors were adjusted at 5×10^11^ vector genomes/ml. Primary oligodendrocytes and neurons were infected by adding 1 µl (5×10^7–8^ vg) to the cell culture medium.

### Antibodies, Plasmids, and Reagents

Antibodies used were as follows: rat PLP (clone aa3), mouse AIP1/Alix (49; BD), mouse Hsc/Hsp70 (Santa Cruz), mouse CD9 (BD Transduction labs), rabbit calnexin (Stressgen), mouse NR1 (Millipore), mouse NR2B (N59/20; Neuromab), rabbit NR3A (Millipore), rabbit GluR3 (Alamone Labs), rabbit GluR4 (Millipore), mouse GFAP (1B4; BD), rat F4/80 [87], mouse NeuN (Chemicon), mouse O4 and mouse O10 [88], mouse Tsg101 (4A10; GeneTex), rabbit GFP (Abcam), mouse αTub (DM1A; Sigma), rabbit TUJ1 (1-15-79; Covance), rat LAMP1 (1D4B; BD), rabbit SIRT2 (Abcam), rabbit hrGFP (Stratagene), rabbit Rab35 (Proteintech), Carbocyanin and HRP secondary antibodies (Dianova), and Alexa secondary antibodies (Invitrogen).

Expression plasmids pPLP-EGFP [42] and pSIRT2-EYFP (this study) were used. SIRT2 sequence was amplified from genomic DNA with PCR (primers 5′-CCGCTCGAGCGATGGACTTCCTGAGGAATTTATTC-3′ and 5′-GGCGAATTCTCTGCTGTTCCTCTTTCTCTTTG-3′) restriction digested and inserted into pEYFP-N1 (Clonetech) between XhoI and EcoRI restriction sites. pEGFP-DynK44A was a kind gift from Luise Florin (University Medical Center Mainz, Department of Medical Microbiology).

Reagents used were as follows: NMDA, AMPA, MK801, NBQX, bicuculline (Tocris), GW4869, Dynasore, Methyl-β-Cyclodextrin, Cytochalasin D, PKH67, PKH26, glutamate, D-serine (Sigma), Pitstop2 (Ascent Scientific), and Alexa568-Transferrin (Invitrogen).

### Transfections, RNAi, RT-PCR, and qPCR

Transient transfections of Oli-neu cells were performed by electroporation using the Bio-Rad Gene Pulser X-Cell [89]. pOL were transfected with Rab35 siRNA (Dharmacon L-042604-01) using Amaxa technology (Lonza) and the Amaxa Nucleofector Kit, Primary Neurons (program O-005). Transfection of pOL with NR1 siRNA (Dharmacon L-045931-01) was carried out using Lipofectamine RNAiMAX (Invitrogen). Exosomal RNA was isolated from 100,000× g pellets using miRNA high affinity kit (Roche). cDNA was synthesized using the Quantitect reverse transcription kit (Qiagen), and Cre and Pgk1 were amplified by PCR. Total brains derived from E10, E14, P0, P7, and adult C57Bl/6N mice were homogenized using TissueRuptor (Qiagen). Total RNA was prepared with the miRNeasy kit (Qiagen), cDNA was synthesized, and qPCR was performed with a StepOne instrument utilizing TaqMan assays recognizing MOG, NG2, and normalizers Pgk1 and β-actin (Applied Biosystems).

### Exosome Isolation and Analysis

Culture supernatants were collected from Oli-neu cells (between div 1–3) and pOL (between div 5–8) and exosomes were isolated by differential centrifugation and flotation density gradient centrifugation as described before [10] with modifications. Briefly, culture supernatants of pOL or Oli-neu cells were collected and cleared from debris by successive centrifugation for 10 min at 60× g and for 20 min at 10 000× g (4°C). Membrane particles remaining in the supernatant were pelleted by ultracentrifugation for 1 h at 100,000× g and 4°C using the SW40 rotor (Beckman). For analysis, the pellet was resuspended in SDS-PAGE sample buffer and subjected to 12% or 10% SDS-PAGE and Western blotting. For nanoparticle tracking analysis, 100,000× g pelleted exosomes were resuspended in PBS and analyzed using the Nanosight LM10 system. To prevent aggregation of exosomes and loss of material due to tube adherence, membrane particles were centrifuged onto a 200 µl sucrose cushion (1.8 M in TBS) for 1 h at 100,000× g and 4°C. For neuronal uptake studies, the cushion including floating exosomes was diluted to a final concentration of 250 µM sucrose and applied to CN. For further purification of exosomes, the diluted cushion was loaded on top of a continuous gradient (0.3–1.8 M sucrose in TBS) followed by centrifugation for 16 h at 100,000× g and 4°C.

### Stimulation and Quantification of Exosome Release

pOL (5×10^6^) differentiated *in vitro* for 7 d were washed with HBSS. Glutamate (50, 100, 200 µM), NMDA (100 µM), or AMPA (100 µM) was added to the cells in Sato 1% HS and reapplied for further 1 h. To inhibit or potentiate glutamate-mediated exosome release pOL were preincubated with MK801 (5 µM), NBQX (25 µM), D-serine (100 µM), or EDTA (5 mM) for 5 min. Exosome pellets and cell lysates were prepared and analyzed as described above. For determination of relative exosome release, x-ray films were scanned and PLP Western blot signals were quantified using ImageJ software (National Institutes of Health). The amount of exosomal PLP was normalized to total cellular PLP. If ER contaminations occurred, the values obtained were further normalized to levels of the ER protein calnexin.

### Cell Lysates and Western Blotting

Cells were scraped in 10 mM Tris pH 7.4, 150 mM NaCl, 1 mM EDTA, 1% Triton X-100, and protease inhibitor cocktail (Roche complete) or PMSF on ice. Nuclei were pelleted by centrifugation for 10 min at 300× g. Cell lysates and exosome samples were subjected to 10% or 12% SDS-PAGE and were blotted onto a PVDF membrane, which was blocked with 4% milk powder/0.1% Tween in PBS. Proteins were detected by sequential incubation of the membrane with primary and HRP-coupled secondary antibodies and developed with enhanced chemiluminescence reagents (Pierce).

### Immunocytochemistry, Immunohistochemistry, and Electron Microscopy

Immunocytochemical staining of cells was performed as described [85]. For immunohistochemical analysis, adult mice were fixed by transcardial perfusion with 4% formaldehyde (Serva), and brains were cut into 30 µm slices on a vibratome and stained according to standard protocols. Fluorescence images were acquired using a fluorescence microscope (DM6000, Leica) or a confocal laser scanning microscope (Zeiss, Axiovert LSM 710) and processed with ImageJ software (National Institutes of Health). Immunoelectron microscopy was performed as described [85]. Briefly, adult mice were fixed by perfusion with 4% formaldehyde (Serva) and 0.2% glutaraldehyde (Science Services) in 0.1 M phosphate buffer containing 0.5% NaCl. Slices of spinal cord and gelatin-embedded pieces of optic nerves were infiltrated in 2.3 M sucrose in 0.1 M phosphate buffer overnight; pieces from the area of the dorsal column and optic nerve samples were mounted onto aluminum pins for ultramicrotomy and frozen in liquid nitrogen. Ultrathin cryosections were prepared using a cryo-ultramicrotome (UC6 equipped with a FC6 cryobox, Leica). Sections were incubated with primary antibodies followed by protein A-gold (15 nm) and analyzed with a LEO EM912 Omega (Zeiss). Digital micrographs were obtained with an on-axis 2048×2048-CCD camera (TRS). For quantitation of multivesicluar bodies, cryosections of optic nerves from three different animals were immunolabeled with anti-PLP and protein A-gold (10 nm). Ten large images were taken from every sample at 8,000× magnification each covering an area of 11.5 µm×11.5 µm, summing up to a total area of 1,322.5 µm^2^ per sample. On every image, the number, location, and labeling of MVBs were analyzed and the number of axons determined. The number of MVBs is expressed relative to the number of axons in the imaged field.

After differential centrifugation exosomes derived from 12×10^6^ cells were pelleted directly on a 75 meshes Ni-Grid, fixed with 4% PFA, and analyzed by EM after washes with a. dest. and embedding in 2% methylcellulose with 0.4% uranyl acetate.

### Transwell Co-culture Assay

Oligodendrocyte/neuron co-cultures were performed in Boyden-Chambers, which allow contact-free culture while permitting exchange of particles through 1 µm pores in a filter membrane (six-well companion plates (353502), six-well cell culture inserts (353102), BD Falcon). Cells growing on filter membranes in the culture inserts (top well) were manipulated, while effects on exosome release or exosome uptake were studied on cells growing in the companion plates (bottom well). To study the effect of neuronal activity on oligodendroglial exosome release, CN were placed on top of pOL and 20 mM KCl or 60 µM bicuculline was added to the top well. After 20 min (bicuculline) or 3 times 2 h (KCl), exosomes were isolated from the supernatant of the bottom well by differential centrifugation and analyzed by Western blotting.

For investigation of exosomal transfer between oligodendrocytes and neurons Oli-neu cells expressing PLP-EGFP and Sirt2-EYFP from plasmids or pOL were grown on top of CN for 1–3 d. pOL were stained with the dye PKH67 according to the manufacturer's instructions and extensively washed, or infected with AAV/MBP-Cre. In the latter case, CN were infected with a recombinant AAV1/2 virus carrying a flox-stop-hrGFP cassette (AAV/CBA-floxstop-GFP) as reporter construct for the detection of Cre activity [46]. PKH67 fluorescence or reporter gene expression in neurons was visualized by fluorescence microscopy or Western blotting and quantified using ImageJ.

To examine neuronal uptake of exosomes by endocytosis, CN and HT22 cells were pre-treated with inhibitors for 30 min (Dynasore, 50 or 100 µM; Pitstop2, 30 µM; CytochalasinD, 10 µM; Methyl-β-Cyclodextrin, 500 µM) and subsequently co-cultured for 24 h with pOL and Oli-neu cells, respectively. Exosome transfer was assessed by Western blotting or fluorescence microscopy. HT22 transfected with pEGFP-DynK44A were co-cultured with Oli-neu cells ectopically expressing PLP-EGFP and Sirt2-EYFP or stained with PKH26 for 24 h. As positive control for endocytosis Alexa568-transferrin (Alexa568-Tf) was used. HT22 cells expressing pEGFP-DynK44A were incubated with Alexa568-Tf for 24 h and analyzed by fluorescence microscopy.

### Microfluidic Chambers

To discriminate between axonal or somatodendritic uptake of exosomes, microfluidic chambers with a microgroove length of 150 µm were used (Xona, standard neuron device 150 µm, Cat# SND150). CN were plated into the device according to the manufacturer's protocol. After 7 div exosomes of PKH67 stained or AAV/MBP-Cre–infected pOLs were either applied to the somatodendritic or axonal compartment of the device. In each case the opposite compartment was filled with a larger volume creating a hydrostatic pressure, thereby fluidically isolating each chamber. In case of the addition of Cre exosomes, CN were infected with a reporter virus as described above. For quantification of uptake efficiency, recombined hrGFP-positive neurons located with their cell bodies in an area of 100 µm above the microgrooves were counted.

### Stereotactic Injection of Exosomes

Two- to five-month-old male and female ROSA26-lacZ reporter mice were anesthetized with ketamine and xylazin and placed in a stereotactic frame. Exosomes prepared from AAV/MBP-Cre–infected pOL by differential centrifugation and dissolved in PBS were injected in the right hippocampus (coordinates in relation to bregma: anteroposterior 1.8 mm, mediolateral 1.8 mm, dorsoventral 1.8 mm) and cerebellum (coordinates: anteroposterior 6.2 mm, mediolateral 2.0 mm, dorsoventral 2.0 mm). As a control, exosomes prepared from uninfected pOL were injected. A volume of 2 µl was injected using a glass microcapillar and a motorized injection pump (World Precision Instruments) at a constant flow rate of 250 nl/min starting 120 s after injection. To limit fluid reflux along the injection track, the needle was kept in place for an additional 5 min after injection. Mice were sacrificed 14 d after injection and perfused with PFA. Brains were vibratome cut (30 µm slices). LacZ and immunofluorescence staining was performed according to standard protocols.

### Analysis of Neuronal Viability and Cell Stress

In co-culture assays, cortical neurons (CN, 0.7×10^6^/6-well) were cultured for 48 h in neurobasal/B27 medium with primary oligodendrocytes (pOL, 1.25×10^6^/insert) in Boyden-Chambers. After or during the co-culture period, neurons were challenged by oxidative stress or nutrient deprivation, respectively. Oxidative stress was induced by incubation with 25 µM H_2_O_2_ for 1 h, nutrient deprivation by exposure to culture medium lacking B27 supplement for 48 h. In the controls, neurons were grown in the presence of blank inserts containing oligodendrocyte-conditioned medium deprived of exosomes by centrifugation at 100,000× g. Neuronal viability was assessed by the MTT assay: 0.75 mg/ml 3-(4,5-dimethylthiazol-2-yl)-2,5-diphenyltetrazolium bromide (MTT, Sigma) was added to the medium for 2 h. Formazan crystals formed were solubilized in a buffer containing 40% [v/v] dimethyl-formamide (Sigma), 10% [w/v] SDS, and 2% [v/v] acetic acid overnight. The absorbance was measured at 562 nm using a plate reader (Tecan Infinite 200). Cells were stained with MitoCapture-dye to visualize the breakdown of the mitochondrial membrane potential utilizing the MitoCapture Apoptosis Staining Kit (PromoCell). Cells were imaged by fluorescence microscopy (DM6000, Leica).

To incubate neurons with exosomes, exosome-containing supernatants or exosome pellets were prepared. Briefly, supernatants were collected over 24 h from pOL (6×10^6^) cultured in neurobasal medium and depleted from cellular debris by subsequent centrifugation for 10 min at 60× g and 20 min at 10,000× g to generate exosome-containing supernatants. A further round of centrifugation for 1 h at 100,000× g was performed to yield exosome-deprived supernatants that were used as control. Exosome-containing or exosome-deprived supernatants were transferred to CN (0.7×10^6^/6-well) and incubated for 12–14 h before assessment of viability by MTT assay. Exosomes from pOL and HEK293T cells as well as liposomes (Liposome Kit by Sigma, preparation according to manufacturer's protocol) were pelleted by differential centrifugation and resuspended in PBS. The number of containing particles was determined by the Nanosight LM10 system to normalize the number of exosomes and liposomes used for treatment (aprox. 6,000 particles/neuron). Exosomes and liposomes were added to CN 12–14 h before the exposure to oxidative stress and subsequent viability assessment.

### Cell Viability (LDH Assay, PI Staining)

To analyze membrane integrity, pOL (7 d in culture) were incubated for 5 h with glutamate in different concentrations (50, 100, 200 µM, 5 mM) and subjected to LDH assay (Roche). The LDH assay was carried out as described in the manufacturer's protocol. For positive controls, cells were incubated with 10 mM NaN_3_ or with H_2_O_2_ prior to the experiment for 1 h. For PI exclusion, pOL growing on coverslips for 7 d were treated with 100 µM glutamate or 2 mM H_2_O_2_ as positive control for 5 h. Cells were incubated with PI (5 µg/ml, Sigma) for 15 min, fixed for 10 min with 4% PFA in PBS, and treated with RNase (100 µg/ml) for 20 min at 37°C. Cells were stained with O10 antibodies recognizing PLP followed by incubation with α-mouse-Alexa-488 and DAPI. DAPI and PI positive cells were counted and the proportion of PI stained cells was calculated.

### Analysis of Intracellular Ca^2+^-Concentration

pOL were grown in a 96-well plate for 7 d. Cells were incubated with 10 µM Oregon Green 488 BAPTA 1, AM (Molecular Probes) in Sato for 30 min at 37°C and stimulated with 100 µM glutamate or 2 µM ionomycin. Fluorescence was recorded over 1 h using a Tecan Infinite M1000 reader (excitation 494, emission 523).

### Statistical Analysis

Significance was calculated by the nonparametric Wilcoxon test (paired, two-tailed) in cases *n*>5. Analysis was performed with the PASW statistics 18 software (SPSS Statistics, IBM).

## Supporting Information

Figure S1 **Glutamate does not affect oligodendroglial cell viability, but mediates Ca^2+^ influx and particle release.** (A) Phase contrast images of the same living primary oligodendrocyte culture before and after glutamate treatment (100 µM, 5 h). (B) Exposure of primary oligodendrocytes (pOL) to 100 µM, 200 µM, and 5 mM glutamate for 5 h and analysis of membrane integrity by LDH assay. H_2_O_2_- and NaN_3_-treated cells were used as positive controls. Error bars, SEM (*n* = 3; * *p*<0.05; ** *p*<0.01; Student's *t* test). (C) Stainings of pOL with antibodies recognizing PLP, propidium iodide (PI), and DAPI after treatment with 100 µM glutamate compared to untreated controls. Cells stressed with 2 mM H_2_O_2_ were used as positive control. PI- and DAPI-stained cells were counted and the proportion of PI positive cells is depicted (scale bar, 50 µm). (D) Nanoparticle tracking analysis (Nanosight) of 100,000× g pellets derived from glutamate-stimulated cells and controls. (E) Transfection of pOL with Rab35- or control-siRNA and quantification of Rab35 knockdown efficiency. Western blot signals of cellular Rab35 were normalized to actin. Error bars, SEM (*n* = 5; * *p*<0.05; Wilcoxon test). (F) pOL were incubated with Oregon Green 488 BAPTA-1, AM for 40 min followed by administration of 100 µM glutamate, and 2 µM ionomycin. Fluorescence was recorded over 1 h.
(TIF)

Figure S2 **Cultured oligodendrocytes express ionotropic glutamate receptors.** (A) Immunostaining of wild-type or NR1-null pOL differentiated in vitro for 7 d with antibodies against glutamate receptor subunits NR1, NR2B, NR3A, GluR3, and GluR4 (scale bar, 20 µM). (B) Magnification of membrane sheaths from (A) (asterisks; scale bar, 10 µM). (C, D) Oligodendroglial NMDA receptors regulate exosome release. (C) pOL were transfected with siRNA against NR1 or control siRNA. Knockdown efficiency was determined by normalizing Western blot NR1 levels to actin (*n* = 3). (D) Boyden chamber co-culture of pOL derived from conditional NR1 knockout mice and cortical neurons (CN). Oligodendroglial exosome release was determined after depolarization of CN with 20 mM KCl (*n* = 1, pool of six embryos).
(TIF)

Figure S3 **Cell-type-dependent uptake of oligodendroglial exosomes.** PKH67-stained primary oligodendrocytes were co-cultured in Boyden chambers for 2 d with cortical neurons or glial cultures containing microglia, oligodendrocytes, or astrocytes stained with specific markers (red). Neurons were immunostained for Tuj1, microglia for F4/80, oligodendrocytes for O4, and astrocytes for GFAP. PKH67-labelled oligodendroglial exosomes are shown in green. Nuclei are stained with DAPI (blue). Scale bar, 50 µm.
(TIF)

Figure S4 **Hsc70 transfer from oligodendrocytes to neurons.** Boyden chamber co-culture of Oli-neu cells expressing EGFP or Hsc70-EGFP with primary cortical neurons (pCN) for 2 d and Western blot analysis of neuronal lysates using antibodies recognizing the EGFP-tag and Tubulin (Tub, shown as normalization standard). Hsc70 is an exosome-associated marker protein. Hsc70-EGFP is selectively transferred to neurons, while EGFP is not, demonstrating that the transfer is a selective process.
(TIF)

Figure S5 **Neuronal cells internalize oligodendroglial exosomes by endocytosis.** (A) PKH67-stained primary oligodendrocytes (pOL) were co-cultured with cortical neurons (CN) in Boyden chambers for 2 d. Neurons were stained with Tuj1 (red) and the late endosomal/lysosomal marker LAMP1 (blue). PKH67-labelled oligodendroglial exosomes are shown in green. 3D projection of a confocal Z-stack is depicted. (B) Co-culture of CN with PKH67 stained pOL for 24 h in the presence of 50 µM Dynasore or untreated (Maximum projection of a confocal Z-stack; scale bar, 20 µm). (C) Western blot of neuronal lysates after Boyden chamber co-culture with Oli-neu cells expressing Sirt2-EYFP and PLP-EGF. CN were pre-treated with different inhibitors for 30 min (Dynasore, 100 µM; Pitstop2, 30 µM; CytochalasinD, 10 µM; Methyl-β-Cyclodextrin (MβCD), 500 µM) and subsequently co-cultured with Oli-neu cells for 24 h. The amount of internalized exosomes is expressed as relative densitometric signal of exosomal PLP-EGFP and Sirt2-EYFP normalized to neuronal tubulin (Tub) (*n* = 3). (D) Oli-neu cells expressing PLP-EGFP were treated with endocytosis inhibitors (concentrations as described in C), and after 24 h, exosomes were collected from the supernatant and analyzed by Western blotting using antibodies against GFP and CD9. (E–G) Cells of the neuronal line HT22 were pre-treated with endocytosis inhibitors for 30 min and subsequently co-cultured with Oli-neu cells either stained with PKH26 (E+F) or expressing PLP-EGFP and Sirt2-EYFP (G) in Boyden chambers for 24 h. (F) The relative exosome uptake was quantified as the amount of internalized PKH26 positive exosomes (red) normalized to total cell number (DAPI, blue) (scale bar, 50 µm, *n* = 2). (G) Neuronal lysates were analyzed by Western blotting for the presence of oligodendroglial exosome proteins SIRT2 and PLP. (H) HT22 cells either expressing dominant negative dynamin (pEGFP-DynK44A) or EGFP as control (pEGFP-c1) were co-cultured with Oli-neu cells expressing PLP-EGFP and Sirt2-EYFP in Boyden chambers for 24 h, and cell lysates were analyzed by Western blotting. (I) HT22 cells expressing EGFP-DynK44A or EGFP were co-cultured with PKH26 stained Oli-neu cells in Boyden chambers for 24 h. As a control pEGFP-DynK44A bearing cells were incubated with Alexa568 transferrin (Alexa568-Tf). Exosomes/Alexa568-Tf are displayed in red, pEGFP-DynK44A/pEGFP-c1 in green, DAPI in blue (scale bar, 20 µm). (J) Quantitative analysis of the experiment described in (I). The amount of internalized exosomes was normalized to total cell number (*n* = 3).
(TIF)

Figure S6 **Oligodendroglial exosomes contain RNA.** (A) Total RNA was prepared from Oli-neu cells and released exosomes and analyzed by agarose gel electrophoresis and ethidium bromide staining. RNase treatment was performed to proof the nature of the nucleic acid. (B) Purification of exosomes by sucrose density gradient centrifugation and quantification of RNA present in the individual fractions. Western blot analysis of the corresponding gradient fractions with antibodies recognizing the oligodendroglial exosome marker PLP revealed that the highest amount of RNA is recovered from exosome fractions. (C) Size distribution of RNAs isolated from exosomes examined by Bioanalyzer technology. Oligodendroglial exosomes lack 18 S and 28 S rRNA. The broad size range is consistent with the presence of miRNAs and mRNAs.
(TIF)

Table S1 **Exosome-associated enzymes.** Exosome-associated enzymes and chaperones identified by proteomics performed with density-gradient purified exosomes isolated from primary oligodendrocytes (adapted from Krämer-Albers et al., 2007 [10]).
(DOC)

## Abbreviations

AAV: adeno-associated virus
AMPA: α-amino-3-hydroxy-5-methyl-4-isoxazolepropionic acid
CN: primary cortical neurons
CNP: 2′, 3′-cyclic nucleotide 3′-phosphodiesterase
CNS: central nervous system
CSF: cerebrospinal fluid
div: days in vitro
GABA: γ-aminobutyric acid
hrGFP: humanized renilla green fluorescent protein
LDH: lactate dehydrogenase
MBP: myelin basic protein
MTT: 3-(4,5-dimethylthiazol-2-yl)-2,5-diphenyltetrazolium bromide
MVB: multivesicular body
NBQX: 2,3-dihydroxy-6-nitro-7-sulfamoyl-benzo[f]quinoxaline-2,3-dione
NMDA: N-methyl-D-aspartate
PLP: proteolipid protein
pOL: primary cultured oligodendrocytes
SIRT2: Sirtuin-2
X-gal: 5-bromo-4-chloro-3-indolyl-beta-D-galactopyranoside
