# Supplementary figures and images for: Neurotransmitter-Triggered Transfer of Exosomes Mediates Oligodendrocyte–Neuron Communication
Source: PLoS Biol. 2013 Jul 9;11(7):e1001604. doi: 10.1371/journal.pbio.1001604 (PMC3706306; doi:10.1371/journal.pbio.1001604)

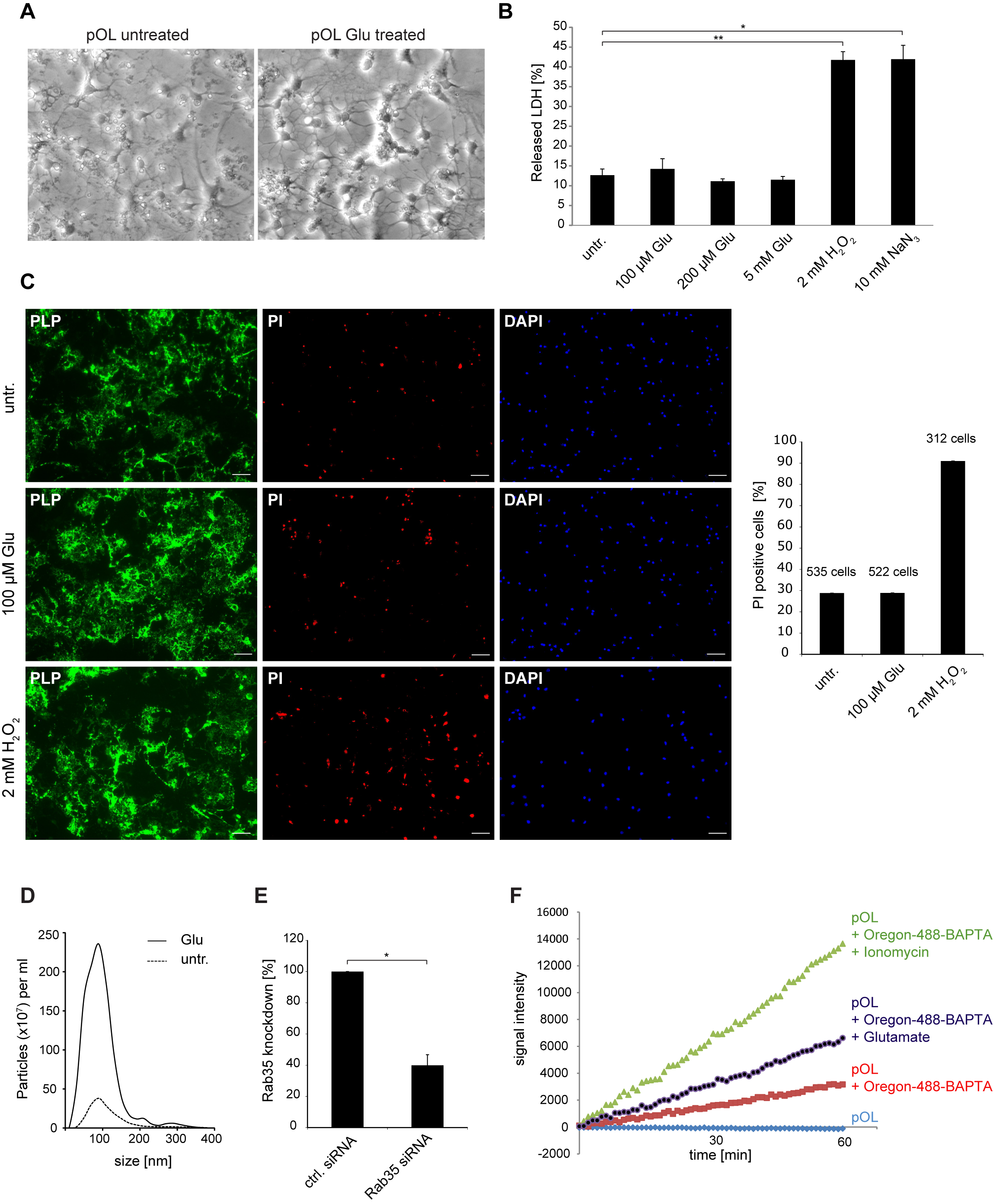

Supplement: Figure S1 — Glutamate does not affect oligodendroglial cell viability, but mediates Ca2+ influx and particle release. (A) Phase contrast images of the same living primary oligodendrocyte culture before and after glutamate treatment (100 µM, 5 h). (B) Exposure of primary oligodendrocytes (pOL) to 100 µM, 200 µM, and 5 mM glutamate for 5 h and analysis of membrane integrity by LDH assay. H2O2- and NaN3-treated cells were used as positive controls. Error bars, SEM (n = 3; * p<0.05; ** p<0.01; Student's t test). (C) Stainings of pOL with antibodies recognizing PLP, propidium iodide (PI), and DAPI after treatment with 100 µM glutamate compared to untreated controls. Cells stressed with 2 mM H2O2 were used as positive control. PI- and DAPI-stained cells were counted and the proportion of PI positive cells is depicted (scale bar, 50 µm). (D) Nanoparticle tracking analysis (Nanosight) of 100,000× g pellets derived from glutamate-stimulated cells and controls. (E) Transfection of pOL with Rab35- or control-siRNA and quantification of Rab35 knockdown efficiency. Western blot signals of cellular Rab35 were normalized to actin. Error bars, SEM (n = 5; * p<0.05; Wilcoxon test). (F) pOL were incubated with Oregon Green 488 BAPTA-1, AM for 40 min followed by administration of 100 µM glutamate, and 2 µM ionomycin. Fluorescence was recorded over 1 h. (TIF) [file pbio.1001604.s001.tif]

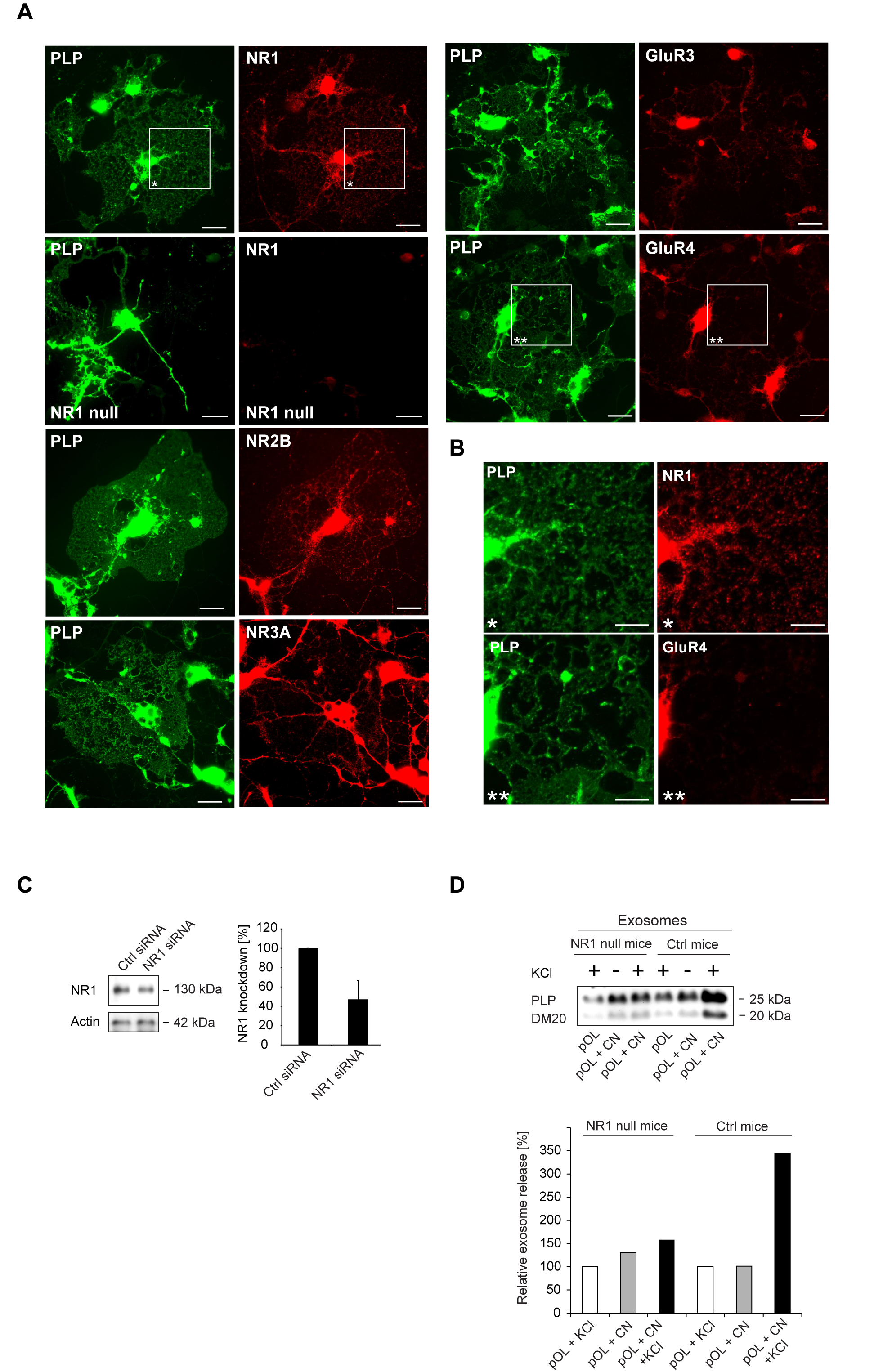

Supplement: Figure S2 — Cultured oligodendrocytes express ionotropic glutamate receptors. (A) Immunostaining of wild-type or NR1-null pOL differentiated in vitro for 7 d with antibodies against glutamate receptor subunits NR1, NR2B, NR3A, GluR3, and GluR4 (scale bar, 20 µM). (B) Magnification of membrane sheaths from (A) (asterisks; scale bar, 10 µM). (C, D) Oligodendroglial NMDA receptors regulate exosome release. (C) pOL were transfected with siRNA against NR1 or control siRNA. Knockdown efficiency was determined by normalizing Western blot NR1 levels to actin (n = 3). (D) Boyden chamber co-culture of pOL derived from conditional NR1 knockout mice and cortical neurons (CN). Oligodendroglial exosome release was determined after depolarization of CN with 20 mM KCl (n = 1, pool of six embryos). (TIF) [file pbio.1001604.s002.tif]

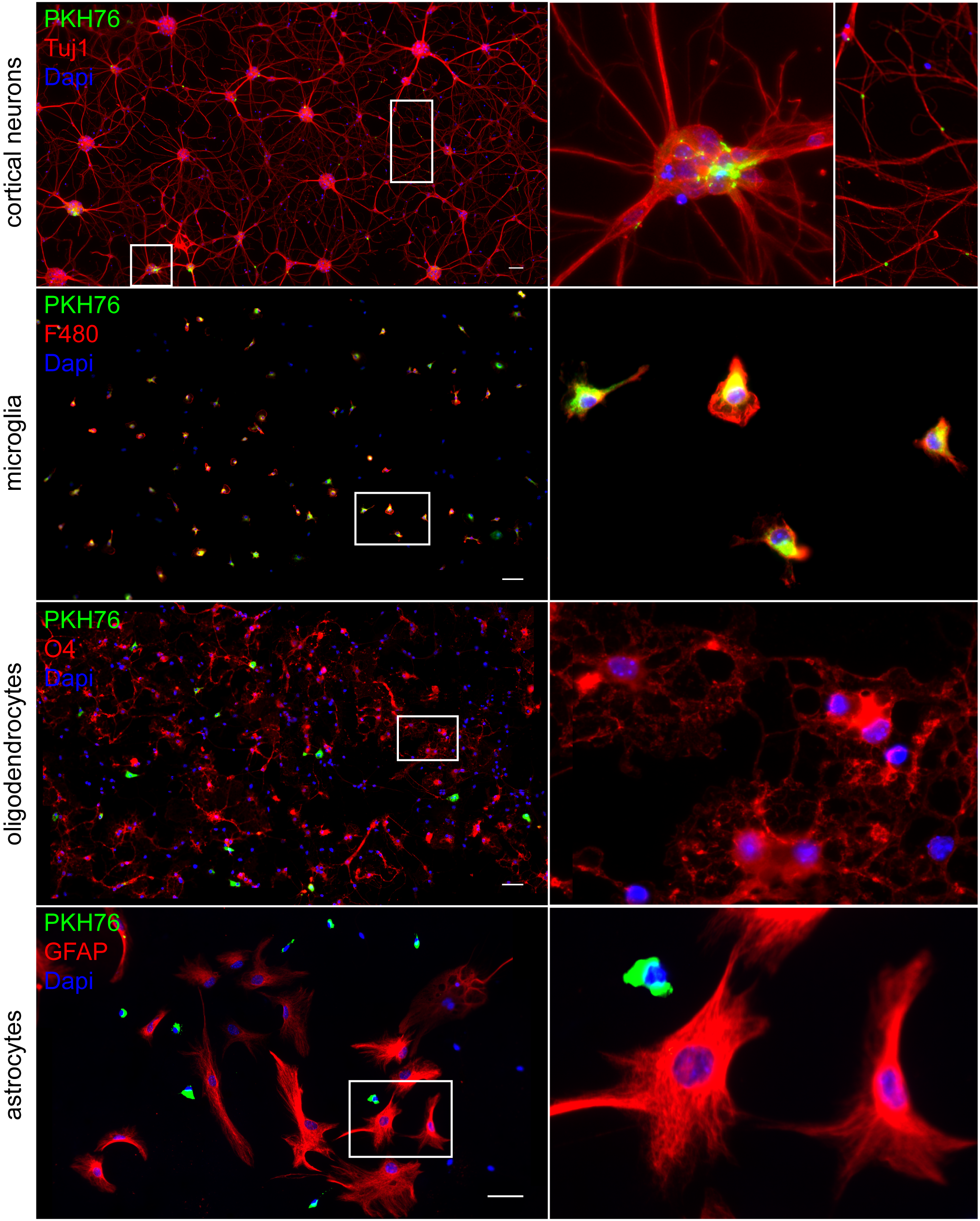

Supplement: Figure S3 — Cell-type-dependent uptake of oligodendroglial exosomes. PKH67-stained primary oligodendrocytes were co-cultured in Boyden chambers for 2 d with cortical neurons or glial cultures containing microglia, oligodendrocytes, or astrocytes stained with specific markers (red). Neurons were immunostained for Tuj1, microglia for F4/80, oligodendrocytes for O4, and astrocytes for GFAP. PKH67-labelled oligodendroglial exosomes are shown in green. Nuclei are stained with DAPI (blue). Scale bar, 50 µm. (TIF) [file pbio.1001604.s003.tif]

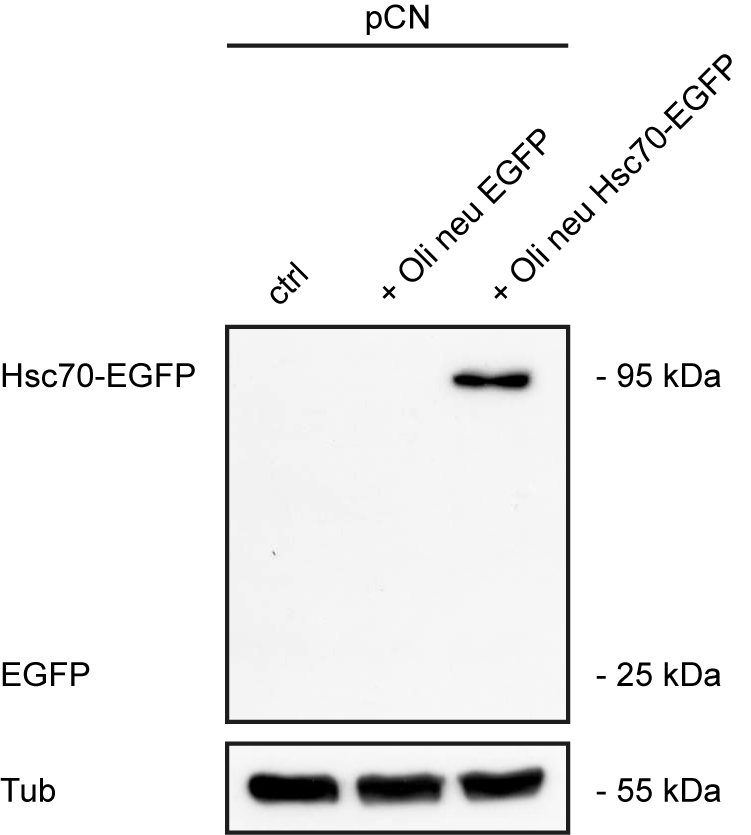

Supplement: Figure S4 — Hsc70 transfer from oligodendrocytes to neurons. Boyden chamber co-culture of Oli-neu cells expressing EGFP or Hsc70-EGFP with primary cortical neurons (pCN) for 2 d and Western blot analysis of neuronal lysates using antibodies recognizing the EGFP-tag and Tubulin (Tub, shown as normalization standard). Hsc70 is an exosome-associated marker protein. Hsc70-EGFP is selectively transferred to neurons, while EGFP is not, demonstrating that the transfer is a selective process. (TIF) [file pbio.1001604.s004.tif]

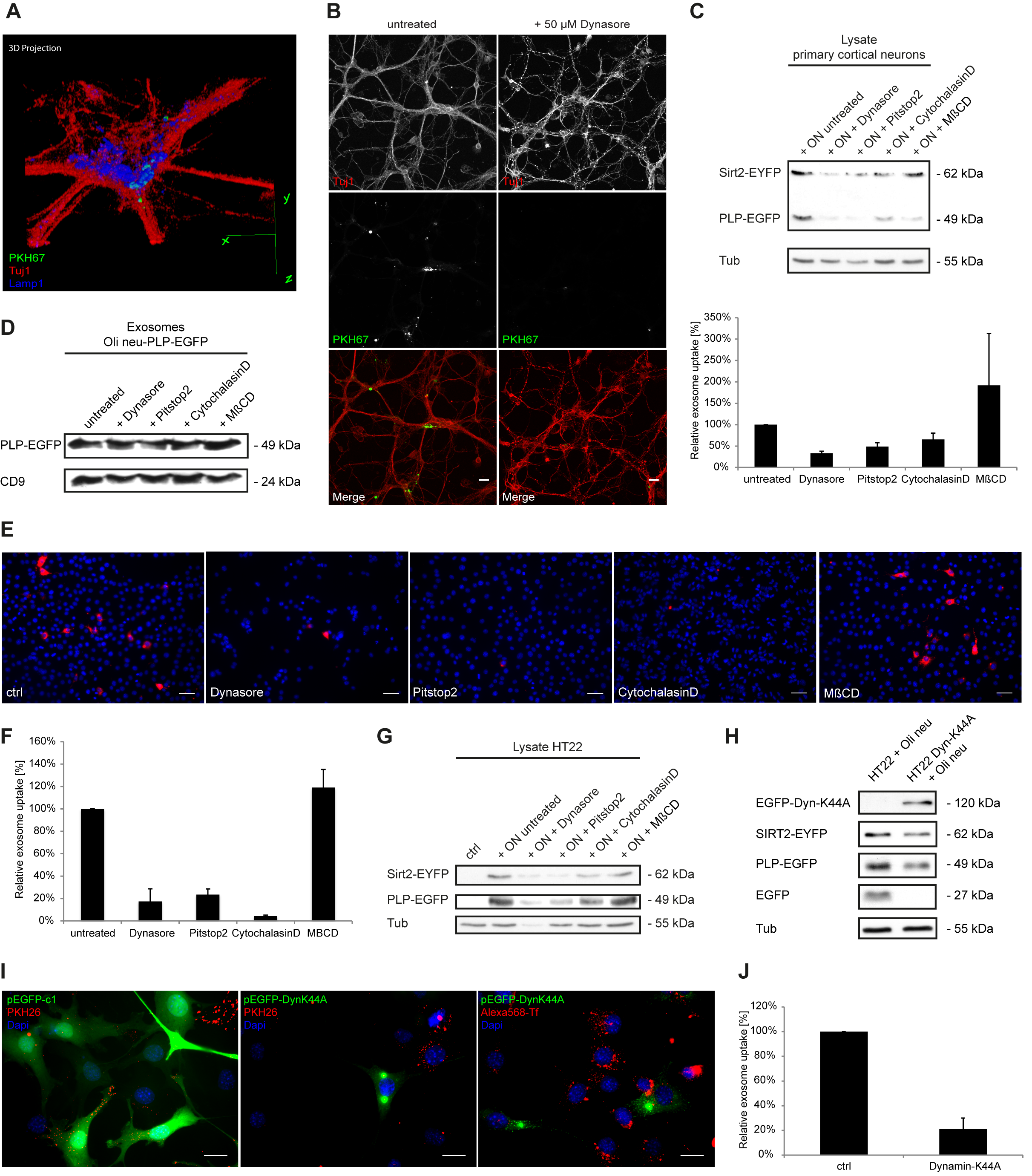

Supplement: Figure S5 — Neuronal cells internalize oligodendroglial exosomes by endocytosis. (A) PKH67-stained primary oligodendrocytes (pOL) were co-cultured with cortical neurons (CN) in Boyden chambers for 2 d. Neurons were stained with Tuj1 (red) and the late endosomal/lysosomal marker LAMP1 (blue). PKH67-labelled oligodendroglial exosomes are shown in green. 3D projection of a confocal Z-stack is depicted. (B) Co-culture of CN with PKH67 stained pOL for 24 h in the presence of 50 µM Dynasore or untreated (Maximum projection of a confocal Z-stack; scale bar, 20 µm). (C) Western blot of neuronal lysates after Boyden chamber co-culture with Oli-neu cells expressing Sirt2-EYFP and PLP-EGF. CN were pre-treated with different inhibitors for 30 min (Dynasore, 100 µM; Pitstop2, 30 µM; CytochalasinD, 10 µM; Methyl-β-Cyclodextrin (MβCD), 500 µM) and subsequently co-cultured with Oli-neu cells for 24 h. The amount of internalized exosomes is expressed as relative densitometric signal of exosomal PLP-EGFP and Sirt2-EYFP normalized to neuronal tubulin (Tub) (n = 3). (D) Oli-neu cells expressing PLP-EGFP were treated with endocytosis inhibitors (concentrations as described in C), and after 24 h, exosomes were collected from the supernatant and analyzed by Western blotting using antibodies against GFP and CD9. (E–G) Cells of the neuronal line HT22 were pre-treated with endocytosis inhibitors for 30 min and subsequently co-cultured with Oli-neu cells either stained with PKH26 (E+F) or expressing PLP-EGFP and Sirt2-EYFP (G) in Boyden chambers for 24 h. (F) The relative exosome uptake was quantified as the amount of internalized PKH26 positive exosomes (red) normalized to total cell number (DAPI, blue) (scale bar, 50 µm, n = 2). (G) Neuronal lysates were analyzed by Western blotting for the presence of oligodendroglial exosome proteins SIRT2 and PLP. (H) HT22 cells either expressing dominant negative dynamin (pEGFP-DynK44A) or EGFP as control (pEGFP-c1) were co-cultured with Oli-neu cells expressing PL [file pbio.1001604.s005.tif]

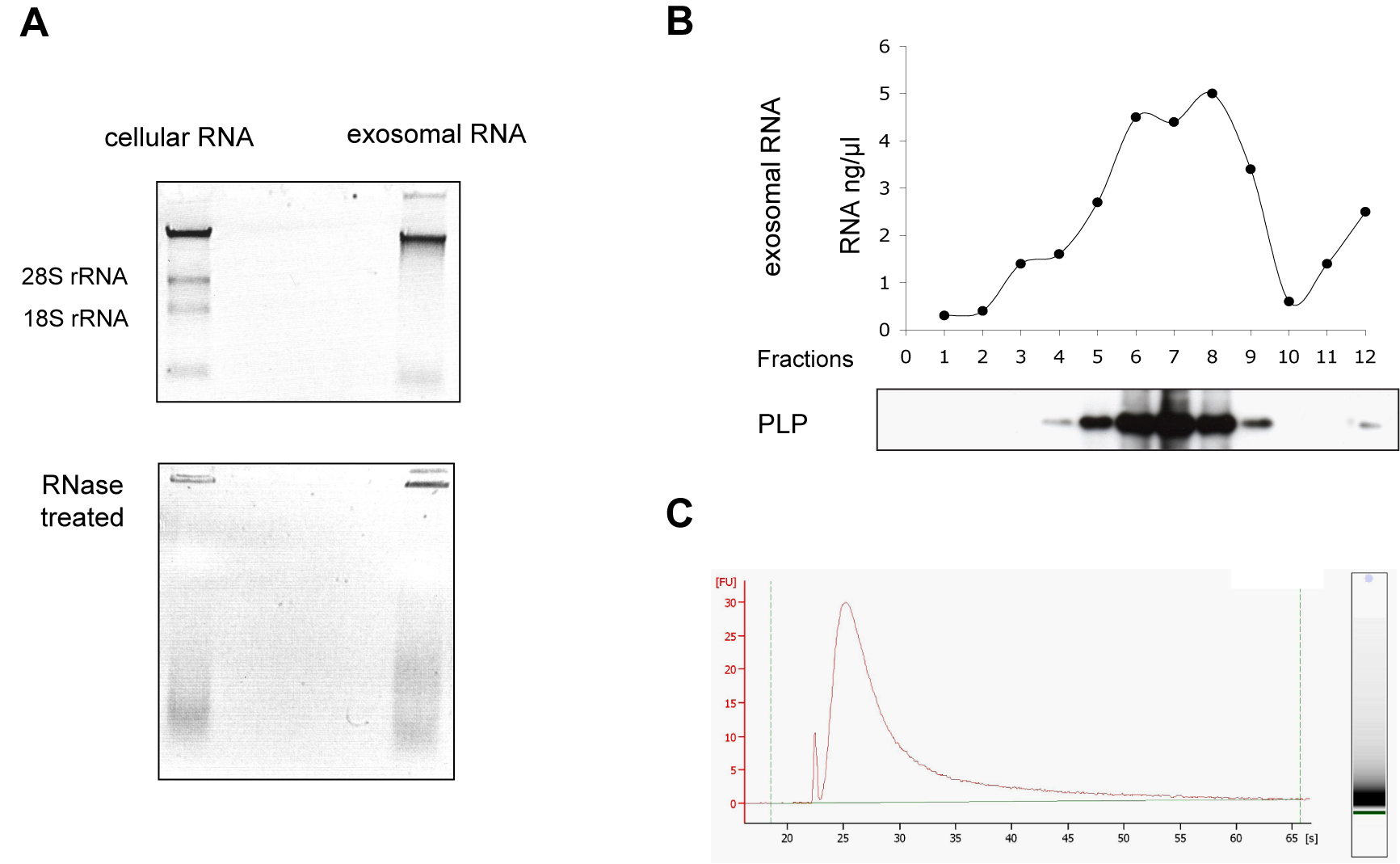

Supplement: Figure S6 — Oligodendroglial exosomes contain RNA. (A) Total RNA was prepared from Oli-neu cells and released exosomes and analyzed by agarose gel electrophoresis and ethidium bromide staining. RNase treatment was performed to proof the nature of the nucleic acid. (B) Purification of exosomes by sucrose density gradient centrifugation and quantification of RNA present in the individual fractions. Western blot analysis of the corresponding gradient fractions with antibodies recognizing the oligodendroglial exosome marker PLP revealed that the highest amount of RNA is recovered from exosome fractions. (C) Size distribution of RNAs isolated from exosomes examined by Bioanalyzer technology. Oligodendroglial exosomes lack 18 S and 28 S rRNA. The broad size range is consistent with the presence of miRNAs and mRNAs. (TIF) [file pbio.1001604.s006.tif]
