# Supplementary material for: Neurotransmitter-Triggered Transfer of Exosomes Mediates Oligodendrocyte–Neuron Communication
Source: PLoS Biol. 2013 Jul 9;11(7):e1001604. doi: 10.1371/journal.pbio.1001604 (PMC3706306; doi:10.1371/journal.pbio.1001604)
Supplement: Table S1 — Exosome-associated enzymes. Exosome-associated enzymes and chaperones identified by proteomics performed with density-gradient purified exosomes isolated from primary oligodendrocytes (adapted from Krämer-Albers et al., 2007 [10]). (DOC) [file pbio.1001604.s007.doc]

**Table S1: Exosome-associated enzymes**

Exosome-associated enzymes and chaperones identified by proteomics performed with density-gradient purified exosomes isolated from primary oligodendrocytes (adapted from Krämer-Albers et al., 2007).

|  | **Accession** | **Entry** | **Description** |
| --- | --- | --- | --- |
|  |  |  |  |
| **Enzymes (Oxidative Stress)** | O08553 | DPYL2_MOUSE | Dihydropyrimidinase related protein 2 |
|  | Q62188 | DPYL3_MOUSE | Dihydropyrimidinase related protein 3 |
|  | P19157 | GSTP1_MOUSE | Glutathione S transferase P 1 |
|  | P35700 | PRDX1_MOUSE | Peroxiredoxin 1 |
|  | Q61171 | PRDX2_MOUSE | Peroxiredoxin 2 |
|  |  |  |  |
| **Enzymes (Metabolism)** | P05064 | ALDOA_MOUSE | Fructose bisphosphate aldolase A |
|  | P00920 | CAH2_MOUSE | Carbonic anhydrase 2 E |
|  | Q8BVI4 | DHPR_MOUSE | Dihydropteridine reductase |
|  | P17182 | ENOA_MOUSE | Alpha enolase |
|  | P16858 | G3P_MOUSE | Glyceraldehyde 3 phosphate dehydrogenase |
|  | P13707 | GPDA_MOUSE | Glycerol 3 phosphate dehydrogenase |
|  | Q04447 | KCRB_MOUSE | Creatine kinase B type |
|  | P52480 | KPYM_MOUSE | Pyruvate kinase isozyme M2 |
|  | P14152 | MDHC_MOUSE | Malate dehydrogenase cytoplasmic |
|  | P15532 | NDKA_MOUSE | Nucleoside diphosphate kinase A |
|  | Q01768 | NDKB_MOUSE | Nucleoside diphosphate kinase B |
|  | P09411 | PGK1_MOUSE | Phosphoglycerate kinase 1 |
|  | P09041 | PGK2_MOUSE | Phosphoglycerate kinase testis specific |
|  | Q8VDQ8 | SIRT2_MOUSE | NAD dependent deacetylase sirtuin 2 |
|  | Q93092 | TALDO_MOUSE | Transaldolase |
|  | Q01853 | TERA_MOUSE | Transitional endoplasmic reticulum ATPase |
|  | P40142 | TKT_MOUSE | Transketolase |
|  | P17751 | TPIS_MOUSE | Triosephosphate isomerase |
|  | P61089 | UBE2N_MOUSE | Ubiquitin conjugating enzyme E2 |
|  |  |  |  |
| **Chaperones** | Q61696 | HS70A_MOUSE | Heat shock 70 kDa protein 1A |
|  | P17879 | HS70B_MOUSE | Heat shock 70 kDa protein 1B |
|  | P16627 | HS70L_MOUSE | Heat shock 70 kDa protein 1L |
|  | P07901 | HS90A_MOUSE | Heat shock protein HSP 90 alpha |
|  | P11499 | HS90B_MOUSE | Heat shock protein HSP 90 beta |
|  | P17156 | HSP72_MOUSE | Heat shock related 70 kDa protein 2 |
|  | P63017 | HSP7C_MOUSE | Heat shock cognate 71 kDa protein |
|  | P17742 | PPIA_MOUSE | Peptidyl prolyl cis trans isomerase A |
|  | Q9CQV8 | 1433B_MOUSE | 14 3 3 protein beta alpha |
|  | P62259 | 1433E_MOUSE | 14 3 3 protein epsilon |
|  | P68510 | 1433F_MOUSE | 14 3 3 protein eta |
|  | P61982 | 1433G_MOUSE | 14 3 3 protein gamma |
|  | P68254 | 1433T_MOUSE | 14 3 3 protein theta |
|  | P63101 | 1433Z_MOUSE | 14 3 3 protein zeta delta |
